# Transcriptomic Adjustment to Decreasing Oxygen Reveals Novel Functional Strategies for Extreme Hypoxia Tolerance in the Copepod *Tigriopus californicus*

**DOI:** 10.1093/gbe/evag013

**Published:** 2026-02-11

**Authors:** Matthew J Powers, Felipe S Barreto

**Affiliations:** Department of Integrative Biology, Oregon State University, Corvallis, OR, USA; Department of Integrative Biology, Oregon State University, Corvallis, OR, USA

**Keywords:** anoxia, glycolysis, trehalose, carotenoids, exoskeleton, *P*_crit_

## Abstract

Hypoxia-induced regulatory changes are well understood across aquatic and terrestrial systems. These changes are normally initiated by elements belonging to hypoxia-inducible factor (HIF) pathway. These elements generate responses that help organisms survive hypoxia, such as protein stabilization, antioxidant activity, or the switch from aerobic to anaerobic metabolism. The HIF pathway is initiated by the transcription factor HIF-α via deactivation of its repressor EGLN. However, recent work revealed that many aquatic invertebrates do not possess HIF-α or EGLN. Among these is the intertidal copepod *Tigriopus californicus*. Although this copepod experiences daily bouts of hypoxia, *T. californicus* tolerates even extended anoxia with minimal mortality. Because *T. californicus* lacks HIF-α, it is unclear how the transcriptional response proceeds on a fine timescale in this species and which physiological strategies they use to cope with severe hypoxia. In this study, we captured gene expression over a species-typical course of hypoxia including normoxia, mild hypoxia (∼3.5 mg O_2_ L^−1^), at critical oxygen tension (*P*_crit_; ∼0.5 mg O_2_ L^−1^), anoxia (0 mg O_2_ L^−1^), and recovery. We identified and clustered genes affected by this hypoxia course and tested for enrichment of Gene Ontology and transcription factor binding site motifs. We identified genes with known responses to hypoxia, including genes with interactions with HIF-α in other systems. We also identified genes related to functions unique to *T. californicus*, including exoskeletal modifications that could represent a specialized response allowing *T. californicus* to persist in extreme hypoxic environments despite lacking HIF-α.

Significance Some species lack genetic elements that normally control the response to low oxygen, or hypoxia, yet must still deal with frequent exposure to minimal oxygen. It is unclear how changes to gene expression progress in these species without these control genes and whether species may resort to specialized and unique biological functions to survive hypoxia. We measured gene expression over a course of hypoxia exposure in one such organism lacking these master control genes, the intertidal copepod *Tigriopus californicus*, and found that this copepod not only still altered the expression of genes involved in a typical hypoxia response in most species, but also genes involved with unique responses that may allow this copepod to tolerate even extreme hypoxia. These results suggest that evolutionary modifications to the genetic hypoxia response can happen in organisms without alterations to canonical downstream responses and without inhibiting novel responses.

## Introduction

The intertidal zone in coastal habitats can be a dynamic and stressful environment because it is subject to cyclical changes based on the daily tides. This includes rapid swings in oxygen content of the water, where during the night, the fauna and flora within pools deplete oxygen via aerobic respiration without the benefit of oxygen replacement from photosynthesis. While pools close to the water line may be refilled with oxygen-rich seawater as the tides rise, splash pools above the water line may experience longer and more intense fluctuations in oxygen content. Any organism that inhabits these pools must be tolerant of extremely low oxygen content and reoxygenation when the sun rises (Legrand et al. 2018; Powers et al. 2025). Our current understanding of the genetic mechanisms underlying hypoxia response in aquatic animals is largely based on taxa inhabiting less stressful environments (Vaquer-Sunyer and Duarte 2008; Rogers et al. 2016), and studies in extremophiles have focused on species of deep ocean oxygen minimum zones (Deutsch et al. 2024). Levels of coastal hypoxia have recently become more intense and widespread, causing mass mortality across several taxa (Whitney 2022), prompting the need to better understand evolutionary mechanisms that allow some taxa to thrive in extreme conditions.

Broadly, animals have evolved two cellular strategies for dealing with hypoxic stress. These are (i) enhanced oxygen delivery to hypoxic tissues by production of new red blood cells (erythropoiesis), increased respiratory proteins, new blood vessels (angiogenesis), and vasodilation and (ii) enhanced energy production via anaerobic pathways, such as glycolysis, while depressing O_2_-dependent OXPHOS (Fuhrmann and Brüne 2017). While both strategies may be used simultaneously in some species, the former occurs primarily in vertebrates and during moderate hypoxia, while the latter is common in invertebrates undergoing periods of prolonged hypoxia or anoxia (Gorr et al. 2006). These physiological responses are ultimately initiated by the hypoxia-inducible factor pathway (HIF) and regulated by the transcription factor HIF-1, which is a protein dimer formed by HIF-α (encoded by *HIF-1α* or *HIF-2α/EPAS1* in vertebrates and *HIF-1α* in invertebrates; Gorr et al. 2010) and HIF-β (encoded by *HIF-β/ARNT*). The formation of the functional HIF-1 heterodimer only occurs when oxygen levels within the cell become critical, and it is controlled by oxygen-sensing enzymes called prolyl hydroxylases (PHDs), primarily PHD2 (or EGLN). Once HIF-1 is active, it works by recognizing and attaching to DNA regions called HREs (hypoxia response elements) located upstream of target genes, thus manipulating their expression patterns (Kaluz et al. 2008).

*HIF-α* and *EGLN* originated early in animal evolution, being found in placozoans and cnidarians (Loenarz et al. 2011; Mills et al. 2018), and the HIF pathway is highly conserved, both in form and function, across animal phyla (Rytkönen et al. 2011; Graham and Presnell 2017). However, recent results by our group (Graham and Barreto 2019; Graham and Barreto 2020) discovered that several species within certain crustacean taxa that are abundant, widespread, and commonly studied have secondarily lost the *HIF-α* and *EGLN* genes and hence likely do not respond to hypoxic stress via the canonical HIF pathway response. These included members of three orders within Cirripedia (Sessilia, Pedunculata, Kentrogonida), such as *Amphibalanus Amphitrite* and *Semibalanus balanoides*, and multiple copepod species across the orders Cyclopoida, Harpacticoida, and Siphonostomatoida, including *Lepeophtheirus salmonis* and *Oithona nana*. This suggests that alternative molecular mechanisms exist for master regulation of cellular response to low oxygen and likely also associated changes in gene networks downstream of master regulators.

The intertidal copepod *Tigriopus californicus* is among species without a canonical HIF pathway, as they lack both *HIF-α* and *EGLN* (Graham and Barreto 2019). Despite this evolutionary loss, this copepod has evolved remarkable tolerance to hypoxic stress and can survive long periods of hypoxia up to 72 h and even 24 h of complete anoxia (Powers et al. 2025). This species inhabits supralittoral splash pools along the west coast of North America, and because *T. californicus* has no pelagic stage, it spends its entire life cycle (nauplius to copepodite to adult) within the same pools (Powlik 1999). Oxygen sensor data from pools on the coast of Oregon, United States, show that *T. californicus* experiences intense selection in the form of extreme hypoxia and even anoxia in the summer months of July through September. These shifts occur after sunset when algae sharing the pools consume oxygen via aerobic respiration and instances of complete anoxia last on average for ≥5 consecutive hours. It is still largely unknown how gene networks normally downstream of *HIF-α* respond in taxa missing the canonical HIF system, including in hypoxia-tolerant species like *T. californicus*.

Previously, differential gene expression was captured in *T. californicus* at three time points: after 3 and 24 h of hypoxia exposure (∼ 0.15 to 0.3 mg O_2_ L^−1^ dissolved oxygen [DO]) and after a full 24-h recovery in normoxic water (Graham and Barreto 2019). This provided a first snapshot of hypoxia gene expression in this system, revealing some expected patterns involving glycolytic activity and mitochondrial function, as well as novel ones. Graham and Barreto (2019) found a strong association between genes involved with exoskeleton components and DO level, highlighting the possibility that chitin metabolism has evolved an important role in an alternative mechanism of hypoxia response.

Our recent work quantifying metabolic variation during hypoxia in *T. californicus* indicated that individual copepods can maintain oxyregulation until extremely low levels of available oxygen and that loss of equilibrium and survival are not affected until the environment is anoxic (Powers et al. 2025). Here we aim to take advantage of this wide range of oxyregulation to characterize transcriptional changes at relatively fine scales of response. We reasoned that physiological breakpoints before oxygen is depleted, such as measured by the respiratory statistic *P*_crit_—the critical oxygen tension at which respiration rate can no longer be controlled by the organism independent of available oxygen in the water—may mark important transcriptomic changes. Similarly, the transcriptomic response to reoxygenation may need to be rapid to keep up with the flood of oxygen in the copepod pools once the sun rises; therefore, capturing gene expression during the initial phases of recovery will also be informative.

To improve our mechanistic understanding of how *T. californicus* responds to hypoxia, we examined the trajectory of gene expression profiles in a course of intensifying hypoxia stress to better predict cellular functions being employed at multiple levels of stress and during recovery. We employed clustering analyses to identify groups of genes with shared patterns of transcriptional change along the DO gradient and tested for enrichment of sequence motifs associated with known transcription factor binding sites. Using multiple approaches for functional annotation, we examined how canonical hypoxia response genes were affected by the exposure course (e.g. those involved with glycolysis and antioxidant roles), but we also were particularly interested in exploring biological functions not previously known to be associated with adaptation to environmental hypoxia as these genes will likely be part of alternative mechanisms of response in lieu of HIF.

## Results and Discussion

**Fig. 1.**
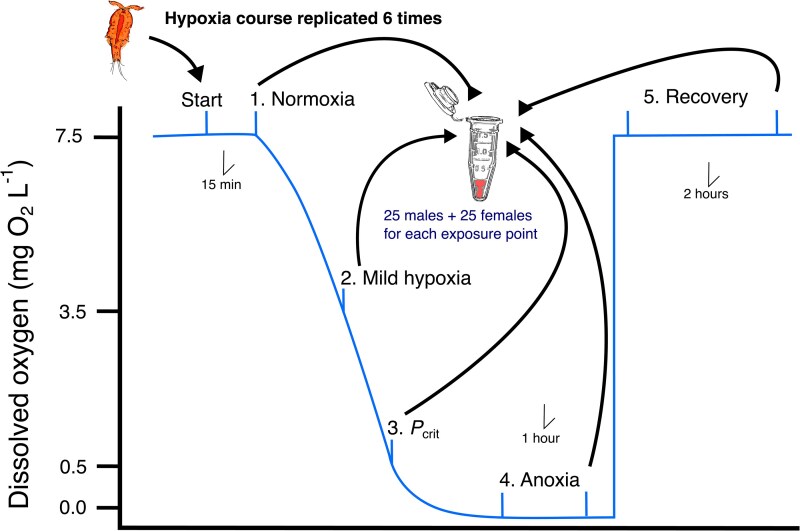
Experimental setup of the hypoxia course. Each exposure level contained 25 male and 25 female copepods. The entire hypoxia course was replicated a total of six times so that each exposure level had a sample size of N = 6 replicates.

We performed mRNA-seq from groups of copepods experiencing intensifying levels of decreasing DO and during reoxygenation (Fig. 1). Exposures were performed in a closed-system microplate respirometer, as in Powers et al. (2025), and pools of copepods were collected at pre-determined DO levels. These were in order: (i) during normoxia, (ii) during mild hypoxia (∼3.5 mg O_2_ L^−1^) halfway through oxygen depletion, (iii) at the species- and population-specific critical oxygen tension (*P*_crit_ = 0.5 mg O_2_ L^−1^; Powers et al. 2025), (iv) after 1-h exposure to complete anoxia (0.0 mg O_2_ L^−1^), and (v) 2 h into recovery after reoxygenation in normoxic water. Sequencing of the 30 libraries (six replicates per treatment) resulted in an average of 14.7 million single reads (range: 9.3 to 17.7 million) and mapping to the *T. californicus* genome resulted in a high rate of uniquely mapped reads across samples (74% to 81%) (Table S1).

**Fig. 2.**
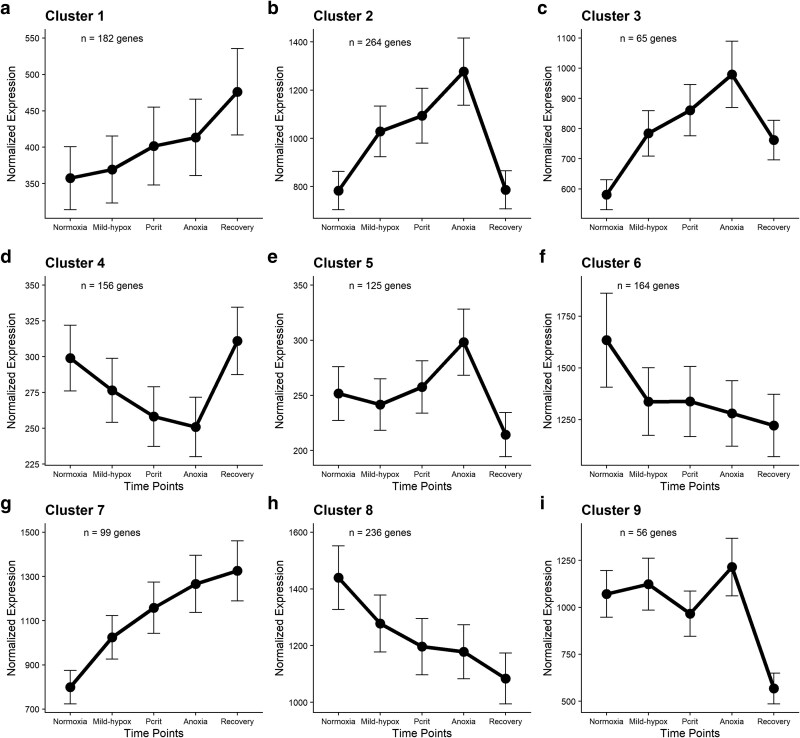
Expression patterns of genes identified to significantly respond over the hypoxia course by maSigPro. Genes were clustered into groups using the default “hclust” (hierarchical cluster analysis) method using Ward.D aggregation and the default *k* = 9 clusters by maSigPro. Black dots are the mean expression at each time point, and error bars represent the standard error of the mean.

Expression data were modeled using regression polynomials with linear, quadratic, and cubic fits using the R package maSigPro v1.74.0 (Conesa and Nueda 2025). Analysis with maSigPro identified 1,347 genes with expression patterns that significantly responded to the hypoxia course and that clustered with other genes (the full list of genes and their *P*-values can be found in Table S2). Hierarchical cluster analysis using *k* = 9 clusters classified these genes into groups with diverse expression patterns encompassing a range of changes over the exposure to and recovery from hypoxic stress (Fig. 2). The number of genes in these clusters ranged from 56 to 264.

### Functional Annotation of Transcription Clusters

We first performed a Gene Ontology (GO) enrichment analysis of the significant genes in each cluster using the R package topGO v2.54.0 (Conesa and Nueda 2025), focusing on biological process (BP) ontology, and passed the results of this analysis to a semantic grouping algorithm using the R package rrvgo v1.14.2 (Sayols 2023) to group enriched GO terms under broad categories of biological functions. Clusters 2, 3, and 5, which captured genes with a transient increase expression as hypoxia intensified, peaking during anoxia, and followed by decreased expression during recovery (Fig. 2), were enriched with genes involved in the metabolic switch from aerobic respiration to anaerobic processes, primarily glycolysis and related sugar pathways (Table 1; Figs. S1 to S3). These included roles in pentose metabolic process and intracellular glucose homeostasis and transport. Across these three clusters also were overrepresented genes related to cell detoxification, mitochondrial function, regulation of apoptosis, lipid metabolism, and bone morphogenetic protein (BMP) signaling pathway regulation (Figs. S1 to S3; Table 1). The BMP pathway does not have a primary role in hypoxia response but has been shown to help mediate oxidative stress during reduced oxygen levels in some cases (Dyer et al. 2014) and may also play a key role cuticle homeostasis in adult invertebrates (Molina et al. 2007; Madaan et al. 2020). Cluster 4, which captured the pattern of expression that is inverse to clusters 2, 3, and 5, was also enriched with genes belonging to many of the same GO terms (Table 1; Fig. S4), and this likely reflects a simultaneous decrease in expression of separate, and perhaps antagonistic, genes involved in these BPs that helps facilitate system-wide responses to hypoxia, such as would be needed with alterations to glucose metabolism during a switch to anaerobic energy production. Cluster 4 was also enriched for genes associated with regulation of the unfolded protein response (Table 1), which is consistent with the need for degradation of misfolded proteins during recovery.

**Table 1:** A summary of select categories taken from the visualization of enriched GO terms created by the “rrvgo” package across all nine maSigPro clusters of genes significantly affected by the hypoxia time course

Broad GO term processes	Clusters enriched for term								
	1	2	3	4	5	6	7	8	9
Immune response	X			X	X	X			
Hormone, steroid, or estrogen regulation	X			X	X			X	
Oxidative stress response	X							X	
Cell detoxification or nitrogen-compound metabolism			X			X		X	X
Glucose homeostasis, glycolysis, and related pathways^a^	X	X	X	X	X			X	X
Mitochondrial-specific targeting and OXPHOS	X				X	X			
Apoptosis	X	X		X		X			
DNA damage repair		X				X			
Unfolded protein response	X			X		X		X	X
Response to external stimuli or stress	X	X		X		X	X		
Heat or cold response	X							X	
Nervous system regulation and retinal response	X	X		X	X		X	X	
Circadian rhythm or clock regulation	X								
Pigment storage									X
Regulation of sperm or egg development	X							X	
Iron ion, calcium ion, or other metabolite homeostasis	X	X		X	X	X			
Lipid or fatty-acid homeostasis	X	X		X		X	X	X	X
BMP signaling pathway					X				

^a^Includes fructose, pentose-phosphate, sucrose and carbohydrate, TCA, and pyruvate pathways.

Several of the functions identified above were also overrepresented in clusters 7 and 8, including carbohydrate and lipid metabolism, unfolded protein response, cell detoxification, and hormone regulation (Table 1; Figs. S5 and S6). Genes in cluster 7 and 8 genes showed a significant unidirectional increase or decrease, respectively, in expression across each level of the hypoxia course. Because expression in these clusters did not reverse toward normoxic levels in the 2 h of recovery following hypoxic stress (as in clusters 2 and 4), it is likely that these clusters include genes involved with aspects of the hypoxia response that cannot be rapidly adjusted or that are important for both survival during hypoxia and during reoxygenation stress. Since the experimental exposure lasted 6 to 7 h, it is possible that clusters 7 and 8 include genes with expression patterns that are influenced by biorhythm. However, clusters 7 and 8 were not explicitly enriched for GO terms associated with circadian function. The lack of a transient response in genes in clusters 7 and 8 should not preclude those clusters from capturing changes in expression due to hypoxia exposure, considering not all gene expression can be altered immediately following the cessation of stress (Kaufer et al. 1998; Sinclair et al. 2007). In the case of hypoxia exposure, modulation of gene expression can be extended into the “recovery” period due to a variety of mechanisms such as increased transcript half-life (Rustad et al. 2013), chromatin modifications (Johnson et al. 2008), or long-term reparative responses to oxidative damage (Fortenbery et al. 2018). We suspect some aspects of recovery from hypoxia by *T. californicus* occur over several hours longer than the 2 h of recovery at which we sampled.

Clusters 1 and 9, characterized by a pronounced change in expression during recovery, were significantly enriched for GO terms associated with reactive oxygen species (ROS) metabolism, wound healing, regulation of glucose metabolism, protein stabilization, promotion of apoptosis, and the regulation of pigment metabolism (Table 1; Figs. S7 and S8). The overlap in overrepresented GO terms among transient and non-transient clusters, such as with glucose metabolism, and the unique enrichment of GO terms in specific clusters, such as BMP signaling or pigment metabolism, suggests *T. californicus* is capable of both a rapid response of repair and detoxification upon the cessation of hypoxia while long-term responses to the stress are simultaneously occurring. Moreover, the processes of hypoxia response start early in *T. californicus*, such as seen in genes of cluster 6 that show a rapid response only during mild hypoxia. Indeed, cluster 6 was also enriched for many of same GO terms as the other clusters, such as cell detoxification and repair, apoptosis regulation, lipid metabolism, unfolded protein response, and mitochondrial function (Table 1; Fig. S9). However, a rapid response to even early stages of hypoxia is not unique to hypoxia-tolerant organisms, such as *T. californicus*. As demonstrated in experiments involving both constant and increasing intensities of hypoxia using the hypoxia sensitive fish, *Galaxias maculatus*, physiological responses to decreasing DO can happen before system-wide shifts in metabolism (Urbina and Glover 2012).

**Fig. 3.**
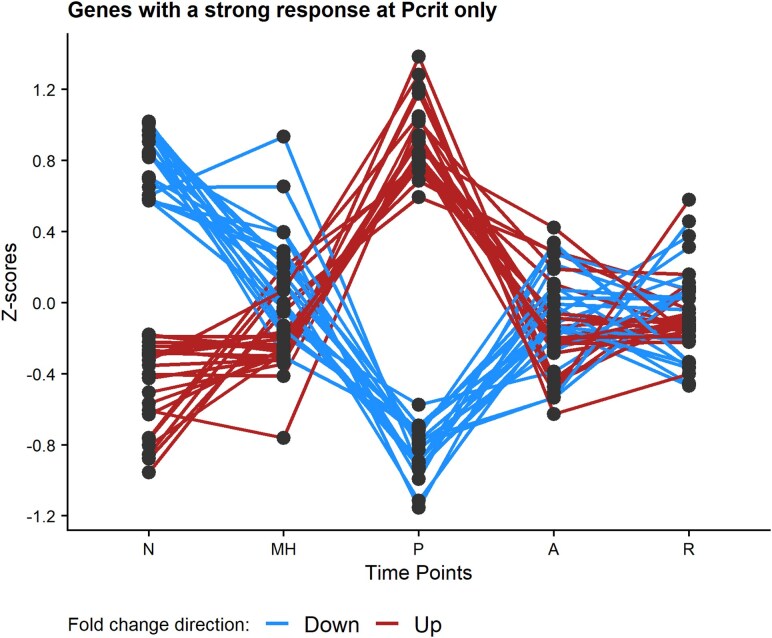
Genes not marked as significant by maSigPro but identified as significantly different in expression between *P*_crit_ and normoxia levels by DESeq2. Gene-standardized z-scores plotted on the *y* axis to show relative change per gene for plotting. Time points are N = normoxia, MH = mild hypoxia, P = *P*_crit_, A = anoxia, and R = recovery.

None of the nine clusters identified by maSigPro displayed a pattern corresponding to a peak change at the *T. californicus*-specific *P*_crit_ of 0.5 mg O_2_ L^−1^. This suggests that physiological changes at the point of critical oxygen tension may affect a smaller subset of genes than can be clustered in large uniform groups of expression patterns. To characterize this transition, we used DESeq2 v1.38.3 (Love et al. 2014 ) to contrast gene expression between pairs of exposure levels with the aim of identifying genes with significant differential expression at *P*_crit_ only (and not present in any maSigPro cluster). This resulted in a subset of 43 significant genes (Fig. 3; Table S3), and this group was enriched for GO terms related to chitin metabolism, glucose and sugar metabolism, and response to ATP (Fig. S10). Several genes in the top ten significant genes in this group were also related to exoskeletal modification (Table S4).

Because descriptions of GO can be very broad, we manually curated lists of genes belonging to known biological functions and pathways to reveal patterns of gene expression and highlight genes from specific clusters of interest. To curate these lists, we mined the Kyoto Encyclopedia of Genes and Genomes (KEGG) for accessions from the *T. californicus* proteome and supplemented these with blastp searches of the *T. californicus* genome using accessions from *Daphnia pulex*, *Drosophila melanogaster*, or, if these were not available, from a member of *Mus* or from *Homo sapiens*. The total number of genes in each manually curated group is listed in Table 2, and the full lists are provided in the public repository at GitHub (https://github.com/mjp0044/Hypoxia-time-series-gene-expression).

**Table 2:** Total number of *T. californicus* genes curated under biological functions of interest

Function or pathway	Total	# significant (maSigPro)	Clusters
Glycolysis	58 genes	15 genes	2, 3, 4, 5, 6, 7, 8
Fructose and mannose metabolism	22 genes	7 genes	2, 4, 6, 8, 9
Starch and sucrose metabolism	54 genes	20 genes	2, 3, 8
Pentose-phosphate pathway	22 genes	8 genes	1, 2, 3, 6, 8
Pyruvate metabolism	40 genes	9 genes	1, 2, 3, 5, 6, 7, 8
TCA cycle	26 genes	2 genes	1, 2
Response to oxygen (GO term)	1022 genes	108 genes	1, 2, 3, 4, 5, 6, 7, 8, 9
Antioxidants	44 genes	12 genes	1, 2, 3, 4, 5, 6, 7, 8
Mitochondria-targeted	613 genes	47 genes	1, 2, 3, 4, 5, 6, 7, 8
Carotenoid and pigment genes	26 genes	10 genes	1, 2, 6, 7, 8
Chitin, cuticle, and exoskeleton	224 genes	52 genes	1, 2, 3, 4, 5, 6, 7, 8

The totals for glycolysis and related pathways (fructose, starch/sucrose, pentose-phosphate, pyruvate, and TCA) also include genes that are known to be present in two or more of these pathways. Likewise, there is probable overlap in the genes in the antioxidant, mitochondria targeted, response to oxygen, and carotenoid pathways.

### Canonical and Unique Responses to Oxidative Stress

When available oxygen is depleted, the production of ROS via mitochondrial oxidative phosphorylation (OXPHOS) complex III occurs during the switch to anaerobic metabolism (Chandel et al. 2000). The production of these compounds continues during the initial phases of recovery when rapid reoxygenation floods the mitochondrial ETS with oxygen (Li and Jackson 2002). The effects of ROS during hypoxia, anoxia, and reoxygenation represent a critical biotic challenge to survival and so it is expected that genes with products functioning within the mitochondria, especially in OXPHOS (Fukuda et al. 2007), and genes with products with antioxidant activity should respond (Liu et al. 2024). Signaling roles of ROS that instigate responses to oxidative damage in hypoxia and during reoxygenation have been reviewed thoroughly by Frederick et al. (2025). We hence examined the patterns across these non-mutually exclusive categories. Of the 613 genes with mitochondrially targeted proteins (MTPs) identified in the *T. californicus* genome (Barreto et al. 2018), 56 showed expression variation across our analyses. The 47 genes identified by maSigPro were distributed across clusters 1 through 8 (Table 2), with the largest number of these genes found in cluster 2. Among antioxidant-related genes, we detected 12 to vary in transcription the hypoxia time course with patterns grouped in clusters 1 to 8 (Table 2).

Work in multiple animal systems has shown that subunits of mitochondrial OXPHOS complexes exhibit cohesive transcriptional changes in response to hypoxic stress (Zhou et al. 2008; Flight et al. 2011; Gracey et al. 2011; Mossman et al. 2016 Nov 14). Similar to Graham and Barreto (2019), we detected only two OXPHOS genes (of 63 annotated) that responded significantly with any pattern, further illustrating that adjustment of this aerobic pathway does not play a role in *T. californicus* response, at least at the transcriptional level. One of these genes, *COX15*, was also one of the two detected in the earlier study (Graham and Barreto 2019), and it responded by increasing expression monotonically over the hypoxia course and into recovery (cluster 7). The product of *COX15* participates in the assembly of OXPHOS complex IV, which is the primary site of proton pumping across the membrane. Accumulation of COX15 during increasing stress and well into the recovery suggests a possible priming strategy of complex IV in preparation for reoxygenation. For this to be the case, it would mean that assembly does not occur until after hypoxia has ceased, especially given the observation that genes encoding OXPHOS proteins themselves do not also increase during hypoxia. Alternatively, increased *COX15* expression may serve as a reparative or stabilizing strategy for existing respiratory complexes (Herwaldt et al. 2018). The lack of transcriptional adjustment to OXHPOS genes during hypoxia by *T. californicus* may explain why this species does not appear to suppress the rate at which it respires as available oxygen declines, even as oxygen approaches anoxia (Powers et al. 2025).

While aerobic OXPHOS does not seem to participate in hypoxia response in *T. californicus*, we observed *AOX1* to have an active role that peaked at anoxia levels (cluster 3). This gene encodes an alternative mitochondrial oxidase which acts as an alternate exit point for electrons during OXPHOS and is predicted to aid in mitochondrial stability during periods of oxidative stress (Maxwell et al. 1999 ). This component is thought to have evolved over periods of fluctuating oxygen in earth's early oxygenation but in most present-day species, *AOX* is absent (Weaver 2019). However, it has been hypothesized that organisms which inhabit dynamic oxygen environments are more likely to retain *AOX* (McDonald and Gospodaryov 2019; Weaver 2019) and this enzyme may be an uncommon mechanism used by copepods to improve survival beyond what is possible through a canonical antioxidant response alone. Despite the buffer that AOX may provide, we still observed significant transcriptional response in several antioxidant genes members of the glutathione system. For example, glutathione reductase (*Gsr*) and two microsomal glutathione S-transferase genes (*Mgst1* and *Mgst3*) peaked in expression during anoxia (clusters 2, 3, 5), while two other *Gst* genes were most active during both normoxic steps (cluster 4). Glutathione peroxidase (*GPX2*) appears to be upregulated incrementally as stress increases and remains so for at least 2 h during reoxygenation (cluster 7). Interestingly, we did not observe significant transcriptional changes in other expected antioxidant enzymes such as superoxide dismutases (SOD) or catalase. These patterns suggest that initial generation of superoxide may be well-managed by a combination of AOX1 activity and constitutive (non-changing) levels of SOD, but that ROS species accumulate during increased hypoxic stress, requiring the use of glutathione for detoxification. Widespread use of glutathione as an antioxidant during oxidative stress exposure has been documented in other copepods as well, including four species within the order Calanoida (Frederick et al. 2025), indicating this antioxidant system may be frequently used by more than just harpacticoid copepods like *T. californicus*.

Alternatively, *T. californicus* may not need to rely on systems like SOD thanks to its colorful adaptation of carotenoid pigmentation across its entire body. Carotenoid pigments are assumed to be both antioxidants and critical precursors to stress and immune responses in marine species (Weaver et al. 2018 Jan 1; Maoka 2020). Out of 26 *T. californicus* genes putatively involved with the modification, transport, deposition, or cleavage of carotenoids, nine were found to be significantly differentially expressed by maSigPro (Table 2). Four of these genes are predicted to encode carotenoid cleaving enzymes, including beta-carotene oxygenases BCO1 and BCO2, whose expression steadily decreased (cluster 8) and the eighth most statistically significant annotated gene in our dataset, *NinaB*, in cluster 1 with genes that increased in expression during recovery. Several cytochrome P450-encoding genes identified by Weaver et al. (2020) as candidate genes that may encode carotenoid-modifying enzymes in *T. californicus* were found to significantly increase in expression either during hypoxia exposure or during recovery. Carotenoids seem to be important in *T. californicus* for buffering against a variety of abiotic stressors (Caramujo et al. 2012; Weaver et al. 2018 Jan 1) and, therefore, the sequestration and metabolism of these pigments may be an important element to bolster defenses against oxidative stress alongside AOX and the antioxidant activity of glutathione.

It should be noted that carotenoid accumulation is known to fluctuate diurnally in *T. californicus* but only in the eyespot, wherein pigment accumulation increases at noon and remains high through dusk as a response to increased photo-stimulation (Martin et al. 2000). Our experiment was initiated late in the morning, just before this period (see Materials and Methods) and, to our knowledge, no study to date has demonstrated body-wide changes in carotenoid content in *T. californicus* throughout the day. Two studies have documented diurnal variation in carotenoid content of other copepod species, but these studies attributed this variation to changes in daily feeding regime and presented contrasting results in terms of whether the species in each study accumulated more carotenoids during the day versus at night (Ringelberg and Hallegraeff 1976; Ishii 1990). Because our experiment took place during a short window of time spanning the middle of the day, we expect the effect of hypoxia and anoxia exposure to produce a stronger signal in gene expression than biorhythm could explain alone.

### Canonical Patterns of and Modifications to Energy Metabolism

**Fig. 4.**
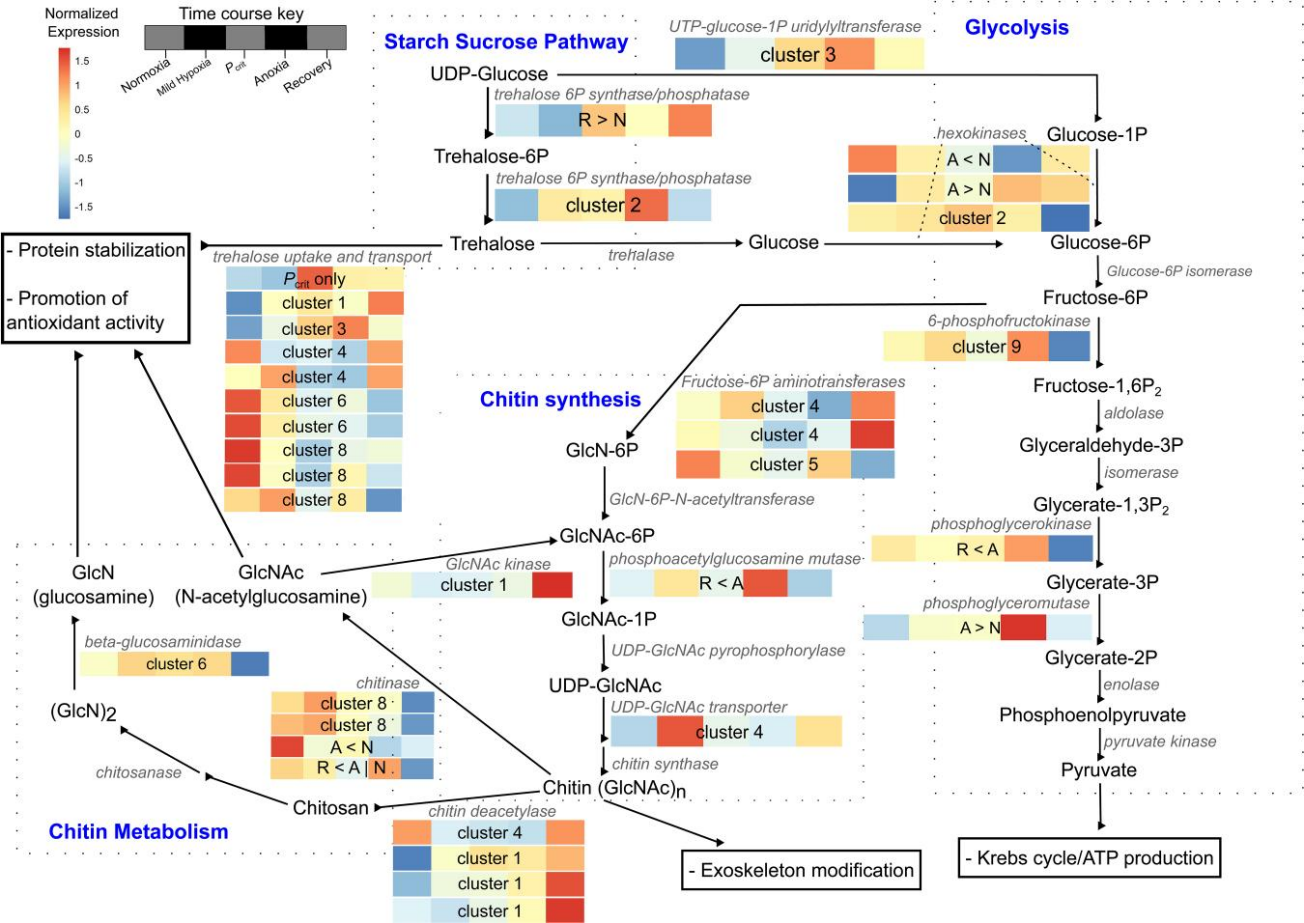
A simplified diagram uniting the starch-sucrose pathway, glycolysis, chitin synthesis pathway, and chitin metabolism and showing genes that significantly responded to the hypoxia course. This diagram is a reduced version of Fig. S10, which shows the entirety of the KEGG pathways for glycolysis, starch-sucrose metabolism, pentose-phosphate metabolism, fructose-mannose metabolism, TCA cycle, and amino sugar metabolism. The heatmaps for significant genes are colored based on gene expression with red indicating an increase and blue a decrease. The five squares correspond to the five hypoxia levels over the exposure course: normoxia, mild hypoxia, *P*_crit_, anoxia, and recovery. Inset on each heat map indicates the cluster or pairwise comparison in which that gene was identified.

When oxygen decreases and is eventually depleted, aerobic organisms often alter their metabolism to rely on the process of glycolysis instead of oxidative phosphorylation (Storey and Storey 2004). GO terms related to glycolysis and intertwined sugar metabolic pathways were enriched in all clusters except clusters 6 and 7 (Table 1), but individual genes involved in these processes were found to be statistically significant and present across all nine clusters (Table 2). To better capture the patterns of change in expression across these related pathways, we combined our manually curated lists of genes for each pathway of glycolysis, starch-sucrose, fructose-mannose, pentose-phosphate, pyruvate, the TCA cycle, and amino sugars with a visualization of expression across those pathways. To do this, we generated KEGG pathways using the ggkegg package v1.4.1 (Sato et al. 2023) in R and overlaid these with the expression heatmaps for all statistically significant genes in our dataset belonging to these pathways. The full pathways can be found in Fig. S11. In Fig. 4, we present a subset of this larger diagram in which we highlight the core of glycolysis, elements of the starch-sucrose pathway related to trehalose, and chitin metabolism. In most organisms, modifications to these pathways to promote anaerobic energy production are a hallmark response coordinated through regulation of HIF-1. Despite the lack of HIF-α, and its repressor, EGLN, in *T. californicus*, this species still mounted a broadly typical and robust transcriptional response to coordinate a switch to anaerobic metabolism during hypoxia exposure (Fig. 4; Fig. S11).

The switch to anaerobic metabolism involves the promotion of glycolysis through the multistep conversion of glucose into pyruvate (Fig. 4; Fig. S11). This requires the upregulation of genes that encode enzymes in the core of the glycolytic pathway as well as downregulation of genes that would normally inhibit glycolysis. We observed the gene *Pepck* transiently increase in expression during hypoxia (cluster 2). This gene encodes phosphoenolpyruvate carboxykinase, which exerts strong control over the rate of gluconeogenesis by converting oxaloacetate to phosphoenolpyruvate interfacing between the TCA cycle and glycolysis (Matsuoka et al. 2015). Concordantly, pyruvate carboxylase (*PC*) increased in expression during anoxia and became elevated even more during early recovery (cluster 1). This is consistent with the need to generate oxaloacetate from the glycolytic pyruvate accumulated during anoxia. Conversion between glucose intermediates and phosphoenolpyruvate also involved canonical upregulation of *Pgk*, *Pfkb4*, and *PGAM2* (Fig. S11). A key inhibitor of glycolysis, *TIGAR* (which encodes a fructose-2,6-bisphosphatase), transiently decreased in expression during hypoxia (cluster 4). *FBP1*, which both participates in glycolysis and has also been shown to inhibit the activity of HIF-α in human carcinomas (Ning et al. 2016), grouped with genes showing an increase in expression during recovery (cluster 1).

Several of the glycolysis genes described above (e.g. *TIGAR*, *Pfkfb4*, and *Pgk*) have each been shown to possess HREs used by HIF-1 in other systems (Ning et al. 2016; Zhang et al. 2016; Maurer et al. 2019; Yang et al. 2024). These were not the only significant genes in our dataset with known interactions with HIF-1, including others whose products do not participate in glycolysis. For example, the protein encoded by *Siah1* stabilizes the activity of HIF-α during the hypoxia response (Fan et al. 2017), and this gene was transiently upregulated during anoxia and was the third most statistically significant gene grouped in cluster 2. The observations that these genes respond in expression as they normally would during the switch to hypoxia-driven glycolysis strengthens the possibility that *T. californicus* has evolved another key regulatory element that can activate many of the same genes regulated by HIF-1. Nevertheless, if switching to glycolysis was enough to persist through severe hypoxia on its own, we would expect other organisms to match the tolerance of *T. californicus* to hours-long bouts of anoxia (Powers et al. 2025). Because *T. californicus* has adapted to become hypoxia tolerant, we speculate that elements of the glycolysis pathway may have been co-opted or integrated into other cellular responses to hypoxia. Indeed, several gene products and intermediate substrates in the glycolysis pathway serve roles across multiple sugar metabolism pathways (e.g. hexokinases) and provide functions outside sugar metabolic pathways themselves (e.g. chitin).

### Sugar Metabolism and Exoskeletal Modification

Like other invertebrates, *T. californicus* relies on the starch-sucrose pathway as a source of sugars for chitin synthesis in the amino sugar pathway. However, elements of the starch sugar pathway can be co-opted for other protective roles and, based on concordance of our results with observations of Graham and Barreto (2019), we speculate that *T. californicus* may take advantage of specific gene products or substrate intermediates to achieve hypoxia tolerance. Specifically, we observed the gene *tpsp*, which encodes a bifunctional trehalose-6-phosphate synthase/phosphatase that can make the disaccharide trehalose, grouped in cluster 2 with genes that transiently increased during hypoxia. Induction of this gene during hypoxia suggests the need for trehalose accumulation. Conversion of trehalose to glucose that can be used for chitin synthesis requires the activity of trehalase, but no trehalase-encoding genes were detected as responding to hypoxic stress in *T. californicus* (Fig. 4). Instead, we observed that ten different trehalose transport genes (*Tret1* or *Tret1-2*) did significantly respond throughout the stress gradient, including one each peaking at the *P*_crit_ and anoxia exposures and two being upregulated during recovery (Fig. 4; Fig. S11). Trehalose and its synthases, like tpsp, are implicated in buffering against hypoxic stress in multiple taxa through a proposed mechanism of protein stabilization and antioxidant activity (Chen and Haddad 2004; Mizunoe et al. 2018). Our findings suggest that *T. californicus* takes advantage of this mechanism to bolster its survival during hypoxia, regulating its use at all stages of exposure and into recovery. Validation of this hypothesis in *T. californicus* requires further investigation and measurement using physiological and metabolic studies.

Graham and Barreto (2019) proposed that *T. californicus* may alter the physiology or morphology of its exoskeleton in response to hypoxia. As noted above, modifications to the exoskeleton involve interconnections between multiple pathways such as the glucose metabolism, starch-sucrose metabolism, and the amino sugar metabolism (Fig. S11). Despite the lack of a transcriptional response in trehalase genes from the starch-sucrose pathway in this study, we still observed other significant regulatory responses by elements of chitin synthesis and amino sugar metabolism (Fig. 4; Fig. S11). Both phosphoacetylglucosamine mutase (*Pgm3*) and UDP-N-acetylglucosamine transporter (*SLC35A3*) were active early in hypoxia, likely providing chitin synthases with the monosaccharide UDP-GlcNAc, a crucial precursor for chitin (Maszczak-Seneczko et al. 2013; Sassi et al. 2014). Concordantly, if chitin synthesis is being utilized during the hypoxic response by *T. californicus*, we would expect that the breakdown of chitin to be downregulated during the hypoxia exposure and upregulated during recovery. We observed this pattern in genes that encode chitinases, which showed steady decreases in expression through recovery, and were highest at the start of the experiment before DO levels decreased (Fig. 4). In addition, genes encoding chitin deacetylases, which breakdown chitin into chitosan, had highest expression during early recovery after mild increases as stress intensified (Fig. 4). These findings are broadly similar to those in Graham and Barreto (2019), although they differ in that we did not detect expression variation in chitin synthase genes, which were expected to be induced as chitinases were downregulated. Nevertheless, chitin synthase gene expression remained stable and, overall, our findings suggest a scenario in which *T. californicus* accumulates chitin during hypoxia to modify its exoskeleton and, following the cessation of hypoxic stress, converts chitin to chitosan, which can be used to buffer against oxidative stress and protein damage during reoxygenation (Mukarram et al. 2023).

Why *T. californicus* may be modifying its exoskeleton to deal with hypoxic stress has yet to be explained. We hypothesize that this copepod may modify its cuticle and chitin content to increase density and decrease porousness to prevent loss of residual intracellular oxygen. This species lacks a circulatory system, respiratory pigments, or gills and passively diffuses oxygen across its cuticle to respire. Rearrangement of the cuticle and the buildup of chitin (to reinforce the exoskeleton or produce chitosan in response to oxidative stress) may allow *T. californicus* to sequester what little oxygen remains when DO in the environment becomes scarce. This strategy would contrast with that of plants, which increase cuticle permeability to facilitate greater gas exchange during hypoxia (Kim et al. 2017). While modification of the exoskeleton has yet to be fully confirmed experimentally via microscopy and advanced imaging, we did observe changes in gene regulation that suggest this process is occurring. Within the top ten most significant genes across all expressions clusters lies the gene *lcc2* (*Laccase-2*, Table S4), which has been implicated in beetles as a critical component of cuticle tanning (Arakane et al. 2005). The process of tanning in invertebrates involves the sclerotization and pigmentation of the cuticle, making it insoluble, hard, and pigmented. Across the hypoxia time course, *lcc2* expression increased in response to hypoxia, grouping with cluster 7 genes. Additionally, the most significantly differentially expressed gene in cluster 2, with genes that displayed transient increases in expression, encodes a cuticle-like protein (Table S4). Other genes predicted to encode cuticle modifying proteins also significantly responded during the time course. These included three genes predicted to encode obst-E proteins, which play a role in chitin binding during cuticle formation (Tajiri et al. 2017), and the another predicted to encode a pro-resilin gene, which helps make the elastic components of the cuticle (Andersen 2010). The obst-E genes were upregulated during hypoxia exposure and the pro-resilin gene was identified to be upregulated at the *P*_crit_ time point compared to normoxia, complementing the enrichment of exoskeleton modifying genes present in the group that solely responded during *P*_crit_. It is possible that the process of cuticle tanning and chitin buildup starts early in hypoxia exposure, becoming critical at the point of critical oxygen tension when *T. californicus* can no longer regulate its respiratory rate with the available DO in the water.

### Transcription Factor Binding Site Enrichment in Gene Clusters

**Fig. 5.**
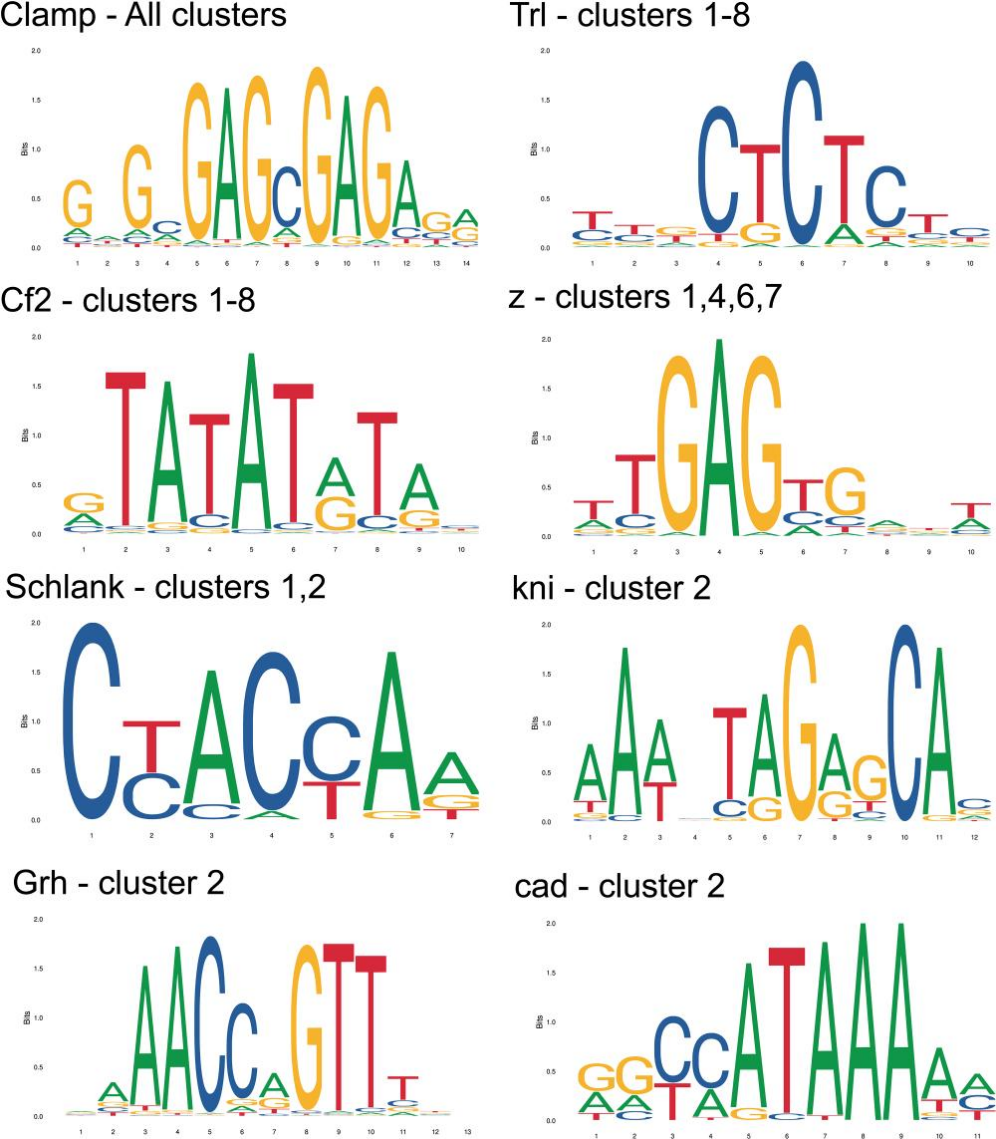
Transcription factor binding site motifs that were commonly overrepresented across promoters of genes in expression clusters. A full list can be found in Table S5.

Following Graham and Barreto (2019), we explored our expression dataset to further identify potential transcription factors participating in the transcription network of hypoxia response in *T. californicus*. We examined each cluster of genes identified via maSigPro for enrichment of known transcription factor binding sites using the MEME suite of tools (Bailey et al. 2015). We used the Analysis of Motif Enrichment (AME) tool to search the upstream promoter regions of genes in each cluster (up to 1,000 bp) for known motifs in the JASPAR database (2022_core_insect_nonredundant). Motifs with significant enrichment were based on default parameters using the adjusted *P*-value from a Fisher's exact text with Bonferroni correction. This analysis revealed 13 motifs across all 9 clusters (Fig. 5; Table S5). The binding site for the transcription factor Clamp, involved in sex-specific alternative splicing and the regulation of chromatin accessibility during exposure to abiotic stress in *Drosophila* (Aguilera et al. 2023), was significantly enriched in all nine clusters. The next most common binding site belonged to Trithorax-like (Trl), present in all clusters except cluster 9. This factor also regulates chromatin accessibility and is connected to proper mitochondrial biogenesis whereby a reduction in Trl function can lead to increased ROS, as shown in *Drosophila* (Dorogova et al. 2023). Like Trl, there was significant enrichment for binding sites of chorion factor 2 (Cf2) in clusters 1 through 8. This factor is associated with the regulation of muscle fiber genes at all stages of life in *Drosophila* (Arredondo et al. 2017); however, its enrichment could also be unrelated to the hypoxia time course and instead could be explained by its association with the circadian gene *timeless*, which was in the top ten most significant genes of cluster 1, thereby indicating at least some changes to gene expression may be due to diel and rhythmic regulation.

Cluster 2, containing genes that transiently increased in expression during hypoxia, was significantly enriched with motifs for three binding sites not enriched in any other cluster. These motifs matched that of knirps (kni), grainyhead (Grh), and caudal (cad) factor binding sites. Knirps-like factors play critical roles in the formation of eggs in *Tribolium castaneum*, mostly through a mechanism of transcriptional repression (Xu et al. 2010), caudal-like factors are known to affect gut homeostasis and increase in expression in response to oxidative stress in *Drosophila* (Choi et al. 2008), and grainyhead-like factors have been shown to influence the integrity and formation of the epithelium, cuticle, and exoskeleton in adult *Caenorhabditis elegans* (Ming et al. 2018; Meeuse et al. 2023). It is possible that grainyhead-like factors also help influence exoskeleton modifications in *T. californicus*, considering previous results that suggest a role of exoskeletal modification to survive hypoxia shown previously (Graham and Barreto 2019) and the identification of several genes involved in chitin and cuticular modification identified in the present hypoxia time course (see previous sections). Future work should aim to experimentally link changes in the expression of key genes, such as *lcc2* mentioned above, with physiological changes to the *T. californicus* exoskeleton confirmed through electron microscopy or micro-CT visualization techniques.

## Conclusions

Our results, based on gene identity and changes in gene expression, indicate that, despite lacking the canonical master hypoxia response regulatory gene, *HIF-1α*, *T. californicus* has evolved a flexible response to hypoxia that facilitates their occupation of a habitat characterized by frequent, extreme fluctuations in available oxygen. While physiological evidence is needed to confirm our hypotheses, we suggest that this is accomplished with minimal adjustment to the expression of genes coding components of mitochondrial OXPHOS complexes except for a possible priming strategy involving the assembly of cytochrome *c* oxidase (complex IV). Rather than suppressing OXPHOS activity in the face of oxidative stress, *T. californicus* mounts a transient antioxidant response using the glutathione system and the alternative oxidase AOX to buffer against ROS and stabilize mitochondrial membranes. These responses are likely bolstered by antioxidant activity of carotenoid pigments, whose cleavage was downregulated steadily through hypoxia and into reoxygenation. While *T. californicus* does not appear to slow its respiration via OXPHOS, the species does transiently increase its usage of anaerobic glycolysis at even the early stages of hypoxia exposure but abandons this strategy quickly during recovery. Components of related sugar metabolism pathways may have been co-opted to buffer against hypoxia exposure, involving adjustments to the usage of trehalose and the chitin byproduct chitosan which may help abate oxidative stress during hypoxia exposure and reoxygenation, respectively. Furthermore, modifications to the exoskeleton of *T. californicus* occur early during hypoxia exposure, and by the time DO levels reach this species’ *P*_crit_, modifications to chitin metabolism and cuticle constituency are widespread. To maintain respiration even with minimally available oxygen, we hypothesize that *T. californicus* increases the density of its exoskeleton to minimize oxygen loss by passive diffusion. While this overall process is not fully reversed, *T. californicus* begins to nullify some of these adjustments during the first hours of recovery. The transient nature of many of these transcriptomic adjustments likely evolved as an adaptation to the fluctuating and stochastic intertidal environment in which this species inhabits.

## Materials and Methods

### Copepod Cultures

Copepods used in this experiment were collected in San Diego, California (32.7333 °N, 117.2500 °W) and were maintained in large, outbred lab cultures in 400-mL beakers for a minimum of 3 months (approx. three generations) prior to any manipulation. This was done to remove the carryover of environmental physiology. The copepods used in this study were further prepared from newly hatched individuals originated from these stock cultures, which allowed us to better control density, water quality, and food availability. To create these experimental subcultures, we removed 240 developed egg sacs from gravid females in the laboratory stock cultures and placed them in new 400-mL beakers to hatch, at a density of 20 clutches per beaker. The experimental cultures were maintained in the same conditions as stock cultures at 20 °C in 35 ppt artificial saltwater (ASW) on a 12h:12 h light:dark cycle in incubators. They were supplied fresh ad libitum mixtures of *Nannochloropsis* and *Isochrysis* microalgae and ground-up spirula wafers and Tetramin fish flakes (Tetra, Blacksburg, VA, United States). We waited until these subcultures were fully mature at a minimum of 30 d old but no older than 44 d old so that all copepods sampled in this study were roughly age matched within 14 d of each other.

### Hypoxia Exposure

We exposed groups of copepods to five treatment levels along a course of hypoxia exposure: normoxia, mild hypoxia, severe hypoxia at *P*_crit_ (critical oxygen tension), anoxia, and recovery (Fig. 1). Copepods used in the experiment were isolated from the age-matched cultures 24 h prior to experimentation and placed in dishes containing clean ASW with no food. A total of 125 males and 125 females were haphazardly divided across the treatment levels with each treatment group containing 25 copepods of each sex (50 individuals total). This procedure was replicated a total of 6 times so that there were *n* = 6 replicates of each exposure level across the hypoxia course (Fig. 1).

On the day of the experiment, the groups of 50 copepods were added to clean, filtered ASW in 500-µL wells of a closed-system microplate respirometer placed in a dark incubator at 20 °C and sealed with air-tight film, a rubber gasket, and a weighted block as recommended by the manufacturer (Loligo Systems, Copehagen, Denmark). Because this respirometer is a closed system, copepods depleted oxygen in the water, eventually creating an anoxic environment after approximately 2 h. This mimics the onset of anoxic conditions that *T. californicus* experiences in the wild on a nightly basis (Powers et al. 2025). After initiating data logging, DO levels were monitored for each well and samples removed at the respective DO level or time point (Fig. 1). Wells from the normoxia treatment were removed after 15 min, while those for the mild hypoxia group were removed when their DO levels were depleted to 3.5 mg O_2_ L^−1^, which represents the halfway point of oxygen depletion in the closed system (Fig. 1). For the severe hypoxia at critical oxygen tension group, we allowed the copepods to deplete DO to 0.5 mg O_2_ L^−1^ before removing them from the well, as this was determined to be the average point at which the San Diego copepods reach *P*_crit_ (Powers et al. 2025). The anoxia group was allowed to deplete oxygen to 0 mg O_2_ L^−1^ and then kept at anoxia for 1 h before removal. Finally, copepods assigned to the recovery group were exposed to anoxia as above but then immediately transferred to dishes containing normoxic ASW, where they were kept for 2 h. The entire run for copepods exposed to the full regime (normoxia through recovery) took approximately 6.25 h (15 min of normoxia, ∼1 h to 3.5 mg O_2_ L ^−1^, ∼1 h to 0.5 O_2_ L^−1^, 1 h of anoxia exposure, and 2 h of recovery time). The six replicate runs were initiated at approximately 10:00 AM each day. We repeated this experiment six times, for a total of 30 individual samples on which we extracted RNA. This experimental design did not include a paired control for each point of oxygen exposure that mimicked the timing of the full experiment, as in (Urbina and Glover 2012), and therefore, our experiment may also capture some effects of natural biorhythm on copepod gene expression.

When each group was removed from their assigned conditions, they were immediately added to 350 µL of TRIzol reagent (Invitrogen) and homogenized with 1-mm zirconia-silica beads (BioSpec) for 25 s at 3,500 oscillations per minute. Homogenates were stored at −80 °C until RNA purification.

### RNA Library Preparation, Sequencing, and Data Processing

Total RNA was purified using Direct-zol RNA MicroPrep Kit (Zymo Research), followed by DNase treatment (TURBO DNase, Invitrogen), and then cleaned using RNA Clean & Concentrator-5 kit (Zymo Research). RNA concentration was estimated via fluorescence (Qubit, Thermo Fisher), and RNA integrity was checked with Agilent 2100 bioanalyzer prior to library preparation. For library preparation, we randomly assigned samples into four groups to minimize the potential for batch effects. The four sets of libraries were prepared on separate days within a span of one week using the QuantSeq 3′ mRNA-Seq V2 FWD Kit unique molecular identifiers (UMI, Lexogen). Number of PCR cycles for enrichment was determined via quantitative PCR, and libraries were then amplified using the PCR Add-on Kit V2 for Illumina (Lexogen) using 13 cycles. Fragment size distribution of each library was checked using Agilent TapeStation High Sensitivity D5000, and they were then pooled at equimolar amounts for sequencing as 100-bp single-end reads on a single lane of the Illumina NextSeq2000 instrument (Oregon State University Center for Quantitative Life Sciences).

Following a quality check using FastQC v0.12.0 (Andrews 2023), reads were trimmed of adapter and UMI sequences using *bbduk* using the following parameters: minlength = 25, trimq = 20, ftl = 10, k = 13, ktrim = r, usesshortkmers = t, mink = 5, and qtrim = r. This retained only reads ≥ 25 nucleotides after trimming the first 10 bases (UMI tags) and any terminal base with quality score < 20. RNA-seq reads were mapped using STAR v2.7.11 (Dobin et al. 2013) against the *T. californicus* reference genome (NCBI Accession: GCA_007210705.1; Barreto et al. 2018), with the following parameters: outFilterMultimapNmax 10, alignIntronMax 10,000, and alignMatesGapMax 10,000. We used featureCounts v2.0.5 (Liao et al. 2014) along with the genome gff file to count uniquely mapped reads on the forward strand within mRNA. We obtained an average of 10.4 million reads counted per sample (range: 7.2 to 11.9 million), which represents an average mapping rate of 77.66% (range: 74.66% to 80.96%) (Table S1).

### Transcriptional Profiling

Processing of count data and differential gene expression analyses were completed in R (R Core Team 2025). Sample counts were assembled into a count matrix using the “read_featureCounts” function from the hciR package v1.7 (Stubben 2024). Prior to normalization using the DESeq2 package v1.38.3 (Love et al. 2014 ) with default parameters, the count matrix was filtered to remove counts of mitochondrial DNA and genes that had fewer than ten reads in at least six samples (which is the lowest number of replicates in the experiment). We also corrected the count matrix for potential batch effects from the multi-day replication of the hypoxia course by using the “Combat_seq” function from the Combat-seq package integrated into the sva package v3.50.0 (Leek et al. 2025).

We identified genes that significantly changed their expression over the hypoxia time course using the maSigPro package v1.74.0 (Conesa and Nueda 2025). This package creates a regression matrix to identify significant changes in expression per gene using the “p.vector” function. We specified the model using with the following parameters: significance threshold Q = 0.05, with MT.adjust = “BH” for Benjamin-Hochberg false discovery rate (FDR) adjustment, and counts = TRUE to use a negative-binomial link function with Θ = 10 because our matrix was in the form of normalized count data. We specified the degree of the regression fit polynomial as “degree = 3,” which models the inclusion of a linear, quadratic, and cubic term. Statistically significant genes were filtered using an r-squared cutoff of 0.1 using the “get.siggenes” function from maSigPro.

We also examined six pairwise comparisons of interest by quantifying differential expression using DESeq2. These contrasts included (i) normoxia versus mild hypoxia, (ii) normoxia versus *P*_crit_, (iii) normoxia versus anoxia, (iv) normoxia versus recovery, (v) anoxia versus recovery, and (vi) mild hypoxia versus anoxia. Differentially expressed genes were considered statistically significant using an FDR-adjusted threshold of α < 0.1. All code used in these analyses are provided in supplemental data and freely available at our GitHub [mjp0044/Hypoxia-time-series-gene-expression].

### Cluster Analysis and Functional Enrichment

Using the statistically significant genes identified using maSigPro, we performed a cluster analysis to group the genes based on their expression patterns over the full hypoxia time course. We used the “see.genes” function in maSigPro to cluster the significant genes using the “hclust” (hierarchical cluster analysis) method with Ward.D aggregation and *k* = 9 clusters. We tested *k* = 6 through *k* = 11 clusters and inspected clusters patterns for biological interpretability (i.e. whether adding or subtracting clusters introduced meaningful patterns vs. creating redundancy or lumping diverse patterns) and evaluated cluster definition and structure using within cluster sum of squares (wss/elbow plot), silhouette width, and the gap statistic. We found that *k* = 9 clusters offered a desirable balance between interpretable patterns and good cluster definition based on wss, silhouette width, and the gap statistic (Fig. S12). Moreover, cluster stability analysis using 100 bootstrapped resamples revealed all 9 clusters had a stability score of 0.68 or above. The stability score ranges from −1 to +1, where values closer to +1 indicate clusters stably reform during resampling.

We performed a GO enrichment analysis in R using the topGO package v2.54.0 (Alexa et al. 2025). We used a custom *T. californicus* GO annotation (available at our GitHub [mjp0044/Hypoxia-time-series-gene-expression]). We tested for overrepresentation among genes in each cluster defined by maSigPro focusing on the BP ontology and with an FDR cutoff of α < 0.05. We further grouped and visualized the GO enrichment results using the rrvgo package v1.14.2 (Sayols 2023), using a reduced similarity matrix with the “Wang” method (Wang et al. 2007) and a threshold of 0.8 for grouping GO terms.

Because GO term labels can be broad and not clearly reflect pathways of interest, we also manually curated *T. californicus* genes expected to be found in specific pathways or functions of interest based on their potential involvement with hypoxia response. Specifically, we categorized *T. californicus* genes involved in the following pathways: oxidative phosphorylation (OXPHOS), glycolysis/gluconeogenesis, starch and sucrose metabolism, fructose metabolism, pentose-phosphate pathway, pyruvate metabolism, citrate cycle (TCA), chitin metabolism, and carotenoid metabolism. Curation of these lists followed approaches similar to Barreto et al. (2015, 2018). First, we mined the well-curated KEGG to establish a list of proteins composing each of the pathways above. In many instances, these pathways were already populated with KEGG accessions from the *T. californicus* proteome, in which case those genes were included in our lists. When a *T. californicus* accession was not found in KEGG, we identified the accession from *D. pulex* or *D. melanogaster* if available, or from a mammal when the arthropods were not listed. Those accessions were then downloaded directly from KEGG. Using these sequences as a custom database, we performed a blastp search with the *T. californicus* proteome as the query, retaining the best match for each query with e-value < 1e^−20^ and alignment length > 60 amino acids. The final total number of genes in each list is shown in Table 2.

### Enrichment of Transcription Factor Binding Site Motifs

We examined each cluster of genes identified via maSigPro for enrichment of known transcription factor binding sites using the MEME suite of tools (Bailey et al. 2015). We used the AME tool to search the upstream promoter regions of genes in each cluster (up to 1,000 bp) for known motifs in the JASPAR database (2022_core_insect_nonredundant). Motifs with significant enrichment were based on default parameters using the adjusted *P*-value from a Fisher's exact text with Bonferroni correction.

## Supplementary Material

evag013_Supplementary_Data

## Data Availability

Illumina read data were deposited in the NCBI Sequence Read Archive (SRA) under BioProject accession PRJNA1252770. All data files used for statistical analyses, the R script generated for this study, and the production of figures and graphs can be found freely online at GitHub (https://github.com/mjp0044/Hypoxia-time-series-gene-expression).
