# Supplementary material for: Transcriptomic Adjustment to Decreasing Oxygen Reveals Novel Functional Strategies for Extreme Hypoxia Tolerance in the Copepod Tigriopus californicus
Source: Genome Biol Evol. 2026 Feb 11;18(2):evag013. doi: 10.1093/gbe/evag013 (PMC12962235; doi:10.1093/gbe/evag013)
Supplement: evag013_Supplementary_Data [file evag013_supplementary_data.zip › Supplement Final.pdf]

Supplement for

Title: Transcriptomic adjustment to decreasing oxygen reveals novel functional strategies for extreme hypoxia tolerance in the copepod *Tigriopus californicus*

Matthew J. Powers[1*], Felipe S. Barreto[1]

[1]Oregon State University, Department of Integrative Biology, Corvallis, OR, USA

*Corresponding author email: powersm3@oregonstate.edu

This file contains the following items:
Tables S1, S4, and S5
Figures S1-S10, S12

Other supplemental files:
Figure S11 is uploaded separately as a high-resolution figure in pdf format
Tables S2 and S3 are uploaded as separate excel documents.

Upon submission, all supplementary files are freely available for review at this Github link:
https://github.com/mjp0044/Hypoxia-time-series-gene-expression

Table S1. Summary of RNA-seq read processing. Sample replicates are listed where C = normoxia, 3.5 = mild hypoxia, 0.5 = P_{crit}, A = anoxia, and R = recovery sampling points.

Sample	Raw reads	Percent retained after trim	Total mapped	Percent mapped	Uniquely mapped	Percent counted
**Average**	14,721,690	91.10%	12,768,055	95.43%	10,377,819	77.66%
**Min**	9,296,007	88.92%	8,519,329	94.16%	7,217,475	74.84%
**Max**	17,740,487	97.48%	15,139,948	96.38%	11,886,378	80.96%
**0.5-1**	15,080,682	90.39%	13,042,446	95.68%	10,559,447	77.46%
**0.5-2**	15,066,384	90.30%	12,866,570	94.57%	10,488,187	77.09%
**0.5-3**	16,352,714	89.18%	13,980,632	95.87%	11,322,965	77.65%
**0.5-4**	14,173,108	90.30%	12,213,780	95.43%	9,937,041	77.64%
**0.5-5**	15,420,160	90.23%	13,246,498	95.20%	10,667,103	76.66%
**0.5-6**	11,043,075	96.17%	10,102,560	95.12%	8,364,220	78.75%
**3.5-1**	14,476,481	91.62%	12,782,737	96.38%	10,377,008	78.24%
**3.5-2**	9,296,007	97.33%	8,519,329	94.16%	7,217,475	79.77%
**3.5-3**	15,640,161	89.78%	13,465,718	95.90%	10,967,343	78.11%
**3.5-4**	14,354,239	89.21%	12,184,688	95.16%	9,904,052	77.35%
**3.5-5**	13,265,079	89.93%	11,363,570	95.26%	9,193,371	77.06%
**3.5-6**	16,113,538	91.66%	14,143,855	95.76%	11,586,855	78.45%
**A-1**	17,740,487	89.52%	15,139,948	95.33%	11,886,378	74.84%
**A-2**	14,881,716	94.52%	13,364,222	95.01%	10,858,084	77.19%
**A-3**	12,735,602	91.48%	11,180,066	95.97%	8,965,773	76.96%
**A-4**	15,890,635	89.06%	13,539,623	95.67%	10,896,969	76.99%
**A-5**	14,137,237	90.34%	12,256,955	95.97%	9,998,329	78.28%
**A-6**	15,499,781	95.17%	14,079,068	95.45%	11,551,500	78.31%
**C-1**	14,184,895	90.98%	12,417,484	96.22%	10,210,518	79.12%
**C-2**	13,418,024	90.03%	11,557,566	95.68%	9,480,287	78.48%
**C-3**	15,671,108	91.27%	13,694,425	95.75%	11,002,387	76.93%
**C-4**	15,725,830	89.66%	13,476,971	95.58%	10,876,383	77.14%
**C-5**	15,201,451	90.24%	13,123,455	95.67%	10,722,031	78.16%
**C-6**	16,714,325	90.60%	14,443,593	95.38%	11,774,587	77.75%
**R-1**	9,764,254	97.48%	9,076,983	95.36%	7,705,652	80.96%
**R-2**	13,633,312	88.92%	11,495,975	94.83%	9,287,748	76.62%
**R-3**	17,051,950	89.04%	14,552,705	95.85%	11,863,513	78.14%
**R-4**	16,607,656	89.34%	14,060,646	94.77%	11,263,233	75.92%
**R-5**	16,630,154	89.01%	14,130,335	95.46%	11,245,707	75.97%
**R-6**	15,880,651	90.23%	13,539,256	94.48%	11,160,431	77.88%

Table S4. Top 10 genes in each maSigPro cluster, arranged by statistical significance, along with possible roles that may relate to hypoxia response and reoxygenation stress during recovery. Additionally, the top 10 significant genes that responded significantly at P_{crit} only.

Cluster	Gene ID (TCAL)	Gene Annotation	Known Functional Role(s) [references listed as superscripts]
**Cluster 1** 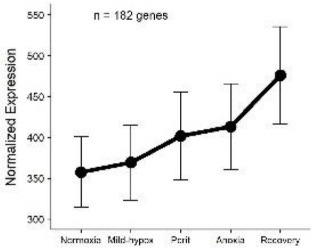	01682	ninaB	Carotenoid dual-oxygenase and isomerase[1]
	12432	hmp Flavohemoprotein	Nitric oxide detoxification and redox homeostasis[2]
	00370	B3GALT1	Biosynthesis of glycoproteins and glycolipids[3]
	04867	SLC13A5	Citrate transport within neurons[4]
	12088	tim (timeless)	Circadian clock and DNA damage checkpoint[5]
	06799	CHAC2	Glutathione degradation for antioxidant homeostasis[6]
	09070	aos (giant-lens)	Photorecepter axon pathfinding and cell determination[7]
	14535	per	Regulation of circadian rhythm[8]
	03517	Mgst3	Cell detoxification via glutathione reactions[9]
	11503	At5g337910	Mitochondrial chaperone[10]
**Cluster 2** 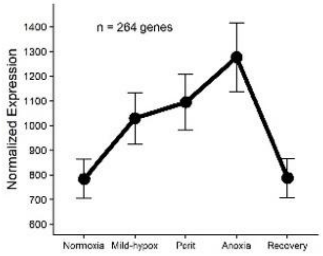	01551	cuticle-like	Exoskeleton modification[11]
	05776	DDO	Deamination of D-aspartate for cell detoxification[12]
	04965	Siah1	Positive inducer of HIF-1α through PHD degradation[13]
	07926	Tmem9	Regulation (+) of Wnt pathway and intracellular pH reduction[14]
	06511	Pyx (pyrexia)	Heat stress response and circadian temperature synchronization[15]
	02126	Gbs-76A	Regulation (-) glycogen binding and (+) glycogen biosynthesis[16]
	03439	Hsp68Bb	Heat shock protein[17]
	10671	Dipetalogastin homolog	Thrombin inhibitor to protect fibrinogen-related proteins[18]
	05534	arrd-17	Responds to osmotic stress and stimulation after starvation[19]
	09693	SPCC1494.01	2-oxoglutarate-dependent dioxygenase[20]
**Cluster 3** 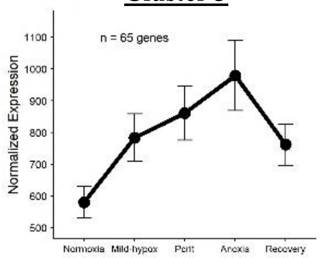	08675	norpA	Phototransduction in photoreceptors[21]
	06802	Venom carboxylesterase-6	Lipolytic activity to generate free fatty acids[22]
	07801	CYP6J1	Oxidoreductase related to cell detoxification (GSH)[23]
	08592	Nrf-6 Nose	Lipid transport[24]
	17390	Slc12a3 homolog	Sodium and chloride homeostasis and receptor for cytokines[25]
	12220	Cyp49a1	Estrogen hormone biosynthesis[26]
	03482	Glutathione reductase (Gsr)	Mitochondrial oxidative stress resistance and cell detoxification[27]
	08955	AHCYL2	Regulation of sodium/bicarb transport and magnesium sensitivity[28]
	07840	UGP2	Generates precursors to glycogen[29]
	08629	Tret1	Trehalose sugar transporter for chitin synthesis[30]

Cluster 4

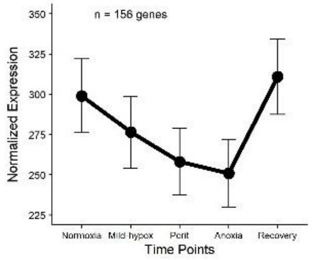

02557	Ets98B	Transcription factor binding during cell differentiation[31]
08167	Ptchd3	Sperm development or function[32]
15032	colt	Gas filling of trachea and epithelial morphogenesis[33]
11065	SIK2	Fatty acid oxidation and glucose metabolism[34]
14798	Gmcl1	Nuclear envelope protein involved in spermatogenesis[35]
04178	Atxn1l	Repressor of Notch signaling[36]
05313	CPPED1	Apoptosis promoter and inhibits glucose uptake in adipocytes[37]
13736	Chitin deacetylase 5	Conversion of chitin to chitosan[38]
00225	mucin-5AC	Extracellular matrix structural constituent of internal epithelia[39]
05545	METTL9	Methylation in mitochondria incl. electron transport subunits[40]

Cluster 5

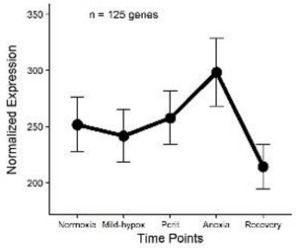

11456	AVT3B	Neutral amino acid transport[41]
10176	TACR2	Receptor for tachykinins (neural, muscular)[42]
02167	nhr-14	Immune regulator and enabler of steroid binding activity[43]
13139	Slc18b1	Translocation of polyamines from cytosol to secretory vesicles[44]
04517	Heat shock 70 kDa cognate 4	Used in protein degradation for healthy embryogenesis[45]
15195	setd7	DNA damage response and heterochromatin organization [46]
16261	Jph3	Forms junctional complexes between plasma membrane and ER[47]
11277	Dat	Dopamine and noradrenaline transport[48]
01071	HMCN1	Influences components of epidermal or epithelial structure[49]
04316	Gls	Glutaminase used in energy prod. and acid-base homeostasis[50]

Cluster 6

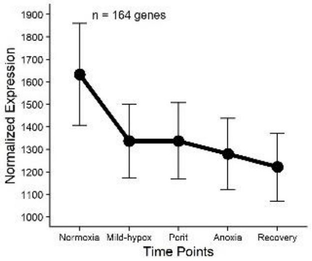

11302	Spns2 (spinster homolog)	Lipid transporter required for immune response[51]
12235	D4Des	Biosynthesis of polyunsaturated fatty acid docosahexaenoic acid[52]
13288	slc46a1	Transport of folate across cell membranes in acidic conditions[53]
07499	Luciferin 4-monooxyganase	Fatty-acyl-CoA biosynthesis for downstream ATP production[54]
00248	CTDSPL2	Regulator of transcription by RNA polymerase II[55]
08165	gst-6 glutathione transferase	Cell detoxification against oxidative stress[56]
01675	Tret1-2 homolog	Trehalose sugar transporter for chitin synthesis[30]
07652	GstD1	Insect glutathione transferase for cell detoxification[57]
12109	NDRG3	Lactate signaling during hypoxia[58]
04108	ATP13A3	Transport of polyamines to the cytosol[59]

Cluster 7

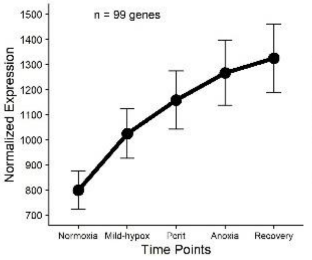

14591	cwo transcription factor	Circadian pacemaker and regulator of Clock gene[60]
08242	Leech-der. tryptase inh. c	Tryptase inhibitor[61]
12804	lcc2 Laccase-2	Used to make exoskeleton insoluble and hard[62]
00766	Slc22a1	Transporter critical for uptake of cations and toxins[63]
05569	lhpp	Metabolizes phosphate and inhibits aerobic glycolysis[64]
05663	fabG	Fatty acid synthesis[65]
11917	SQOR	Detoxifies mitochondria from sulfides for hypoxia resistance[66]
01737	Slc22a7	Hub gene for excretion of toxic anions during hypoxic stress[67]
08982	Soma ferritin	Storage of iron ions during hypoxia[68]
08241	Dipetalogastin homolog	Thrombin inhibitor to protect fibrinogen-related proteins[18]

<table>
<tr><td rowspan="10">Cluster 8
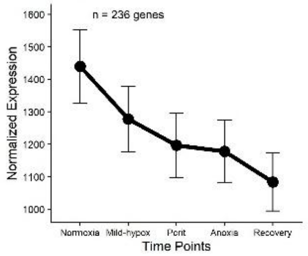
</td><td>13810</td><td>urad</td><td>Purine catabolic processes in the peroxisome[69]</td></tr>
<tr><td>09890</td><td>clpX</td><td>Unfolded protein response in mitochondria[70]</td></tr>
<tr><td>05274</td><td>EHD4</td><td>Membrane reorganization and endosomal transport[71]</td></tr>
<tr><td>11475</td><td>xsc</td><td>Conversion of taurine to sulfite and acetate[72]</td></tr>
<tr><td>01161</td><td>mmlH</td><td>4-methylmuconolactone transporter in aromatics metabolism[73]</td></tr>
<tr><td>09874</td><td>agmo</td><td>Alkylglycerol monooxygenase involved in cuticle stability[74]</td></tr>
<tr><td>07649</td><td>Similar to oxlT</td><td>Uptakes oxalate from the gut[75]</td></tr>
<tr><td>09466</td><td>nad Epmerase dehydratase</td><td>Helps regulate cell surface properties[76]</td></tr>
<tr><td>09716</td><td>Similar to oxlT</td><td>Uptakes oxalate from the gut[75]</td></tr>
<tr><td>12742</td><td>TMEM68</td><td>Alternative triglyceride synthesis and mitochondrial stability[77]</td></tr>
<tr><td rowspan="10">Cluster 9
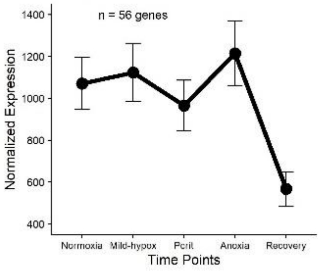
</td><td>02439</td><td>CD63</td><td>Promotes cell survival, cell adhesion, sequestration of pigment[78]</td></tr>
<tr><td>11523</td><td>Venom toxin 11-like</td><td>Secretes OcyC11 like compound affecting cell adhesion[79]</td></tr>
<tr><td>01542</td><td>mdh</td><td>Needed for citric acid cycle and NADH supply to mitochondria[80]</td></tr>
<tr><td>12977</td><td>CG9705</td><td>Cold shock protein needed for mRNA 3'-UTR binding activity[81]</td></tr>
<tr><td>03519</td><td>CG13624</td><td>(+) regulator of transcription involved with TORC1 signaling[82]</td></tr>
<tr><td>05698</td><td>PNKP</td><td>DNA damage repair following oxidative damage[83]</td></tr>
<tr><td>12659</td><td>rost (rolling stone)</td><td>Needed for myoblast fusion during muscle growth and repair[84]</td></tr>
<tr><td>112107</td><td>NA^+/H^+ exchanger beta</td><td>Important for insulin secretion during homeostasis[85]</td></tr>
<tr><td>05177</td><td>Slc12a3 homolog</td><td>Sodium and chloride homeostasis and receptor for cytokines[25]</td></tr>
<tr><td>05281</td><td>XK-related 6-like</td><td>Blood antigen production[86]</td></tr>
<tr><td rowspan="10">P_{crit} cluster
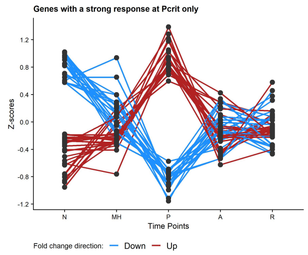
</td><td>16451</td><td>Basic proline-rich isoform X1</td><td>Cell growth[87]</td></tr>
<tr><td>03260</td><td>Sell L-selectin</td><td>Endothelial cell growth[88]</td></tr>
<tr><td>06219</td><td>Mrc2 C-type</td><td>Extracellular collagen modification[89]</td></tr>
<tr><td>09416</td><td>P4HA1</td><td>Key enzyme in collagen synthesis[90]</td></tr>
<tr><td>03107</td><td>resilin pro-resilin</td><td>Cuticle development[91]</td></tr>
<tr><td>11030</td><td>Sc5d Lathosterol oxidase</td><td>Cholesterol biosynthesis and prevention of cell death[92]</td></tr>
<tr><td>06987</td><td>FUCA1</td><td>Fucose-containing glycoprotein degradation[93]</td></tr>
<tr><td>12876</td><td>Compound eye opsin BCRH2</td><td>Photoreceptor activity[94]</td></tr>
<tr><td>09258</td><td>cuticle 7-like</td><td>Exoskeleton modification[95]</td></tr>
<tr><td>05028</td><td>ANKS1A</td><td>Ephrin signaling pathway and neuron pathway regulation[96]</td></tr>
</table>

Table S5. Significantly enriched motifs in the promoter region (maximum 1000bp upstream) of genes in each maSigPro cluster. Within each cluster, motifs are arranged by statistical significance along with possible roles that may relate to hypoxia response and reoxygenation stress during recovery.

Cluster	Motif_ID	Name	*p*-adj	Consensus sequence	Class	Functional role [references listed as superscripts]
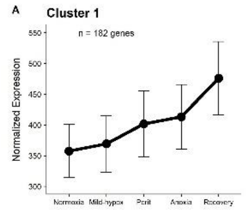	MA1700.1	Clamp	4.43E-07	GWGMGAGCGAGAGR	C2H2 zinc finger	Sex specific alt splicing/regulates chromatin[97]
	MA0205.2	Trl	8.83E-04	RVAAGAGAGAGR	C2H2 zinc finger	Regulates chromatin structure[98]
	MA0255.1	z	4.88E-03	WTGAGTGRDW	Trithorax	transvection-based gene expression[99]
	MA0222.1	exd	1.33E-02	NTTTGACR	Homeo domain	Regulation of neural development[100]
	MA0015.1	Cf2	2.77E-02	RTATATRTAB	C2H2 zinc finger	Follicle cell fate and muscle gene regulation[101]
	MA0237.2	pan	4.21E-02	KCGSCKYBTTTGRT	High-mobility group	Regulation of transcription by RNA pol II[102]
	MA0193.1	schlank	4.23E-02	CYACYAA	Homeo domain	Lipid metabolism[103]
	MA0247.2	tin	4.39E-02	WBTCRAGTGS	Homeo domain	Development of heart and muscle[104]
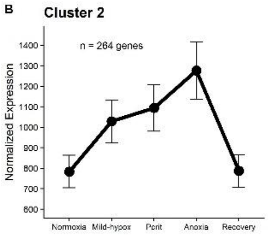	MA1700.1	Clamp	2.03E-10	GWGMGAGCGAGAGR	C2H2 zinc finger	Sex specific alt splicing/regulates chromatin
	MA0205.2	Trl	1.52E-07	RVAAGAGAGAGR	C2H2 zinc finger	Regulates chromatin structure
	MA0015.1	Cf2	9.42E-05	RTATATRTAB	C2H2 zinc finger	Follicle cell fate and muscle gene regulation
	MA1457.1	grh	5.78E-03	VAAACCAGTTTBN	Grainyhead	Epithelial cell and cuticular development[105,106]
	MA0216.2	cad	1.50E-02	RGYMATAAAAM	Homeo domain	Regulation of gut AMP levels[107]
	MA0193.1	schlank	4.01E-02	CYACYAA	Homeo domain	Lipid metabolism
	MA0451.1	kni	5.07E-02	AAWNTAGAGCAS	Nuc rec C4 zinc fingers	Regulates segmentation during development[108]
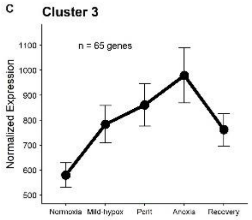	MA1700.1	Clamp	2.59E-08	GWGMGAGCGAGAGR	C2H2 zinc finger	Sex specific alt splicing/regulates chromatin
	MA0205.2	Trl	4.69E-05	RVAAGAGAGAGR	C2H2 zinc finger	Regulates chromatin structure
	MA0015.1	Cf2	5.18E-02	RTATATRTAB	C2H2 zinc finger	Follicle cell fate and muscle gene regulation
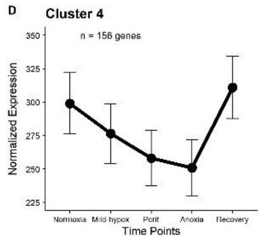	MA1700.1	Clamp	7.79E-08	GWGMGAGCGAGAGR	C2H2 zinc finger	Sex specific alt splicing/regulates chromatin
	MA0205.2	Trl	7.02E-07	RVAAGAGAGAGR	C2H2 zinc finger	Regulates chromatin structure
	MA0015.1	Cf2	2.35E-03	RTATATRTAB	C2H2 zinc finger	Follicle cell fate and muscle gene regulation
	MA0255.1	z	2.56E-03	WTGAGTGRDW	Trithorax	transvection-based gene expression
	MA0247.2	tin	4.72E-03	WBTCRAGTGS	Homeo domain	Development of heart and muscle

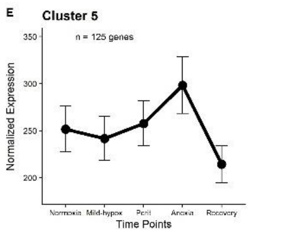	MA0205.2<br>MA1700.1<br>MA0015.1	Trl<br>Clamp<br>Cf2	1.62E-13<br>1.83E-13<br>1.53E-02	RVAAGAGAGAGR<br>GWGMGAGCGAGAGR<br>RTATATRTAB	C2H2 zinc finger<br>C2H2 zinc finger<br>C2H2 zinc finger	Regulates chromatin structure<br>Sex specific alt splicing/regulates chromatin<br>Follicle cell fate and muscle gene regulation
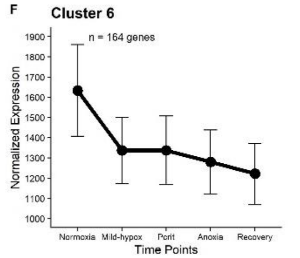	MA1700.1<br>MA0205.2<br>MA0015.1<br>MA0255.1	Clamp<br>Trl<br>Cf2<br>z	3.10E-11<br>3.79E-11<br>9.35E-06<br>3.40E-02	GWGMGAGCGAGAGR<br>RVAAGAGAGAGR<br>RTATATRTAB<br>WTGAGTGRDW	C2H2 zinc finger<br>C2H2 zinc finger<br>C2H2 zinc finger<br>trithorax	Sex specific alt splicing/regulates chromatin<br>Regulates chromatin structure<br>Follicle cell fate and muscle gene regulation<br>transvection-based gene expression
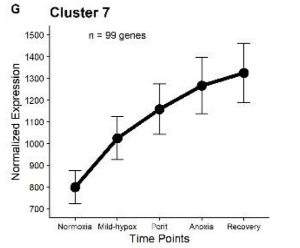	MA0015.1<br>MA1700.1<br>MA0255.1<br>MA0205.2<br>MA0531.1<br>MA0249.2	Cf2<br>Clamp<br>z<br>Trl<br>CTCF<br>twi	1.46E-05<br>1.87E-05<br>5.60E-04<br>5.62E-04<br>2.24E-02<br>3.27E-02	RTATATRTAB<br>GWGMGAGCGAGAGR<br>WTGAGTGRDW<br>RVAAGAGAGAGR<br>CCRMYAGRTGGCGCY<br>RNACACATGTN	C2H2 zinc finger<br>C2H2 zinc finger<br>trithorax<br>C2H2 zinc finger<br>C2H2 zinc finger<br>bHLH	Follicle cell fate and muscle gene regulation<br>Sex specific alt splicing/regulates chromatin<br>transvection-based gene expression<br>Regulates chromatin structure<br>Short and long-range chromatin remodeling[109]<br>Muscle development and Notch activation[110]
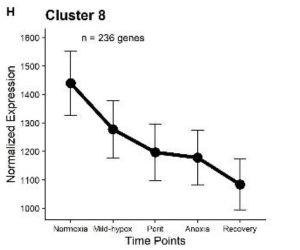	MA1700.1<br>MA0205.2<br>MA0531.1<br>MA0015.1	Clamp<br>Trl<br>CTCF<br>Cf2	1.47E-05<br>2.99E-05<br>1.18E-02<br>5.74E-02	GWGMGAGCGAGAGR<br>RVAAGAGAGAGR<br>CCRMYAGRTGGCGCY<br>RTATATRTAB	C2H2 zinc finger<br>C2H2 zinc finger<br>C2H2 zinc finger<br>C2H2 zinc finger	Sex specific alt splicing/regulates chromatin<br>Regulates chromatin structure<br>Short and long-range chromatin remodeling<br>Follicle cell fate and muscle gene regulation

I 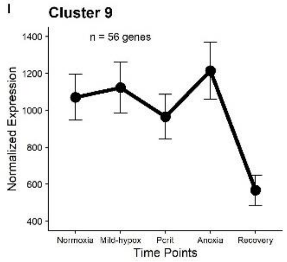	**MA1700.1**	Clamp	4.22E-02	GWGMGAGCGAGAGR	C2H2 zinc finger factors	Sex specific alt splicing/regulates chromatin

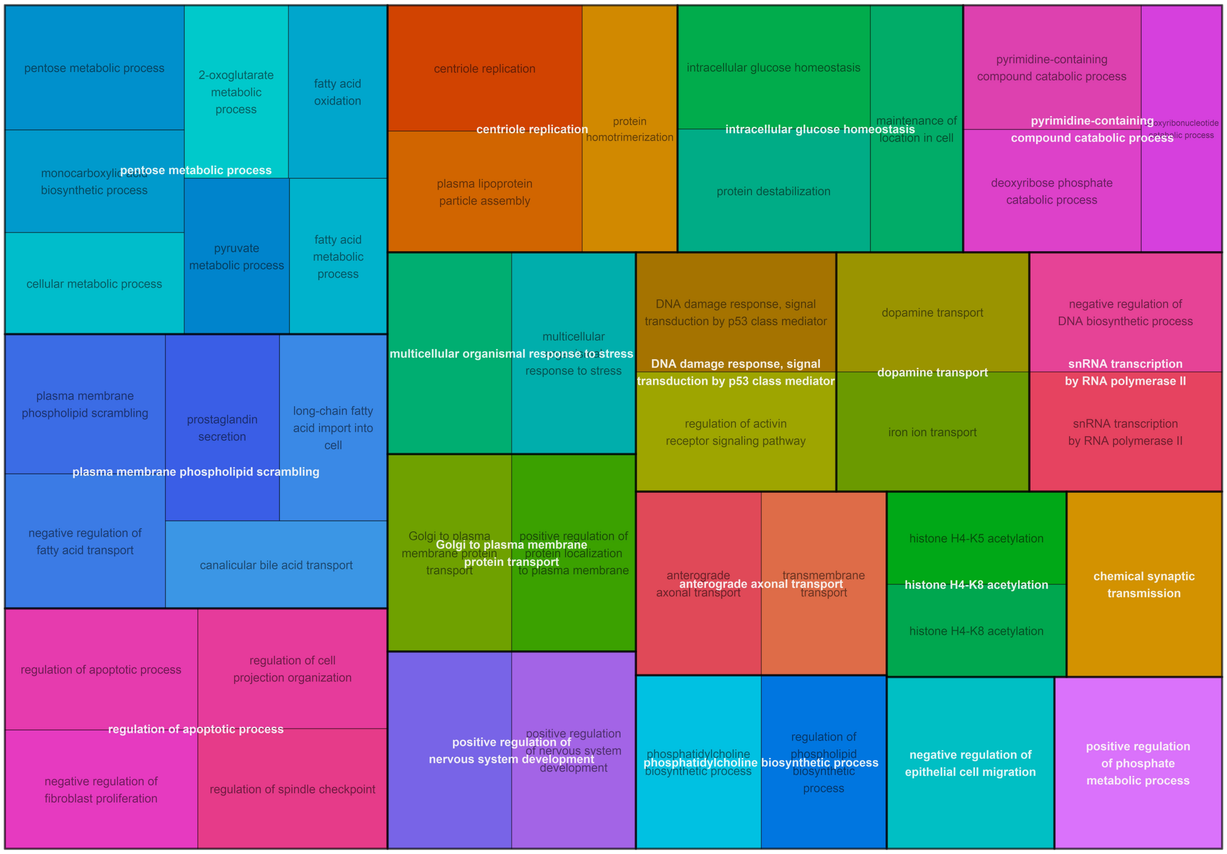

Fig. S1. Cluster 2 treemap from rrvgo reduction and grouping of TopGO enrichment analysis of genes. Genes in this cluster increased expression levels steadily through anoxia and then decreased to pre-stress levels during recovery.

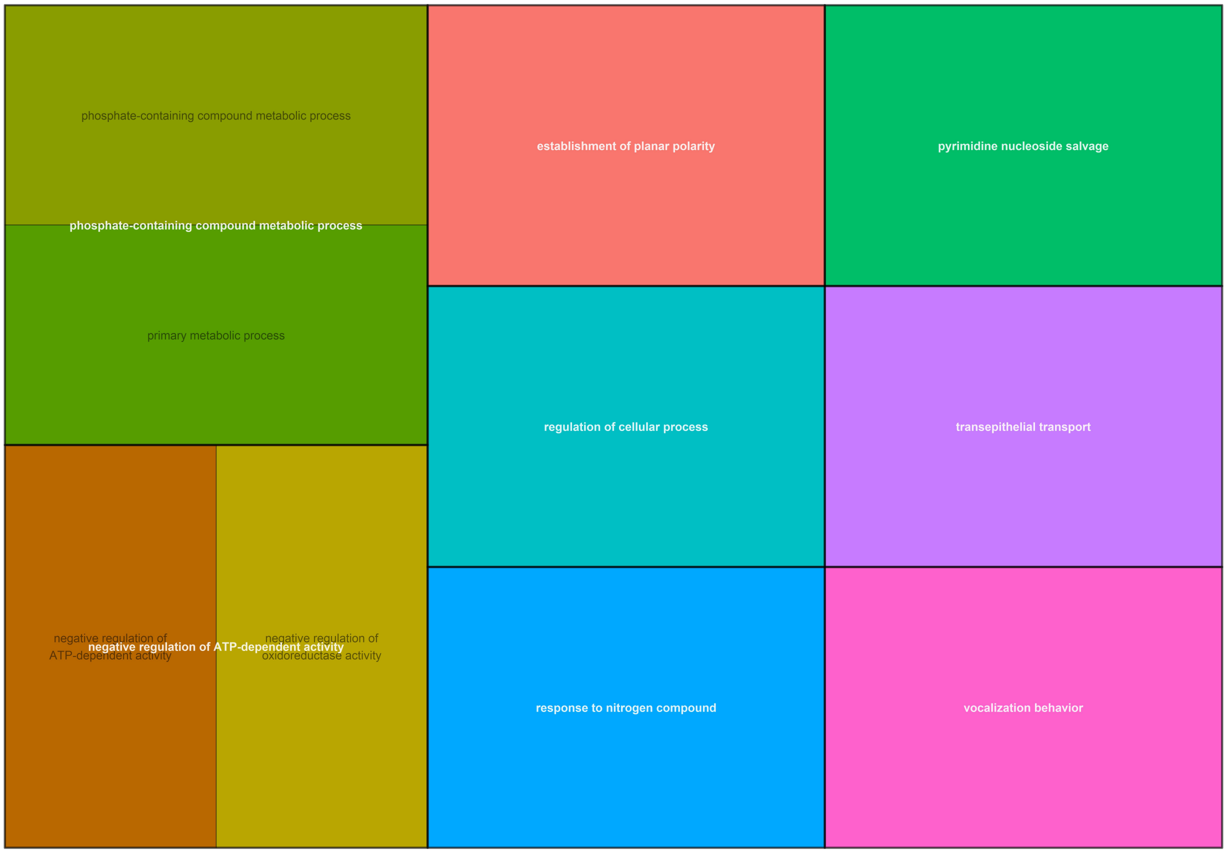

Fig. S2. Cluster 3 treemap from rrvgo reduction and grouping of TopGO enrichment analysis of genes. Genes in this cluster increased expression levels steadily through anoxia and then decreased during recovery but not to the same level as during normoxia.

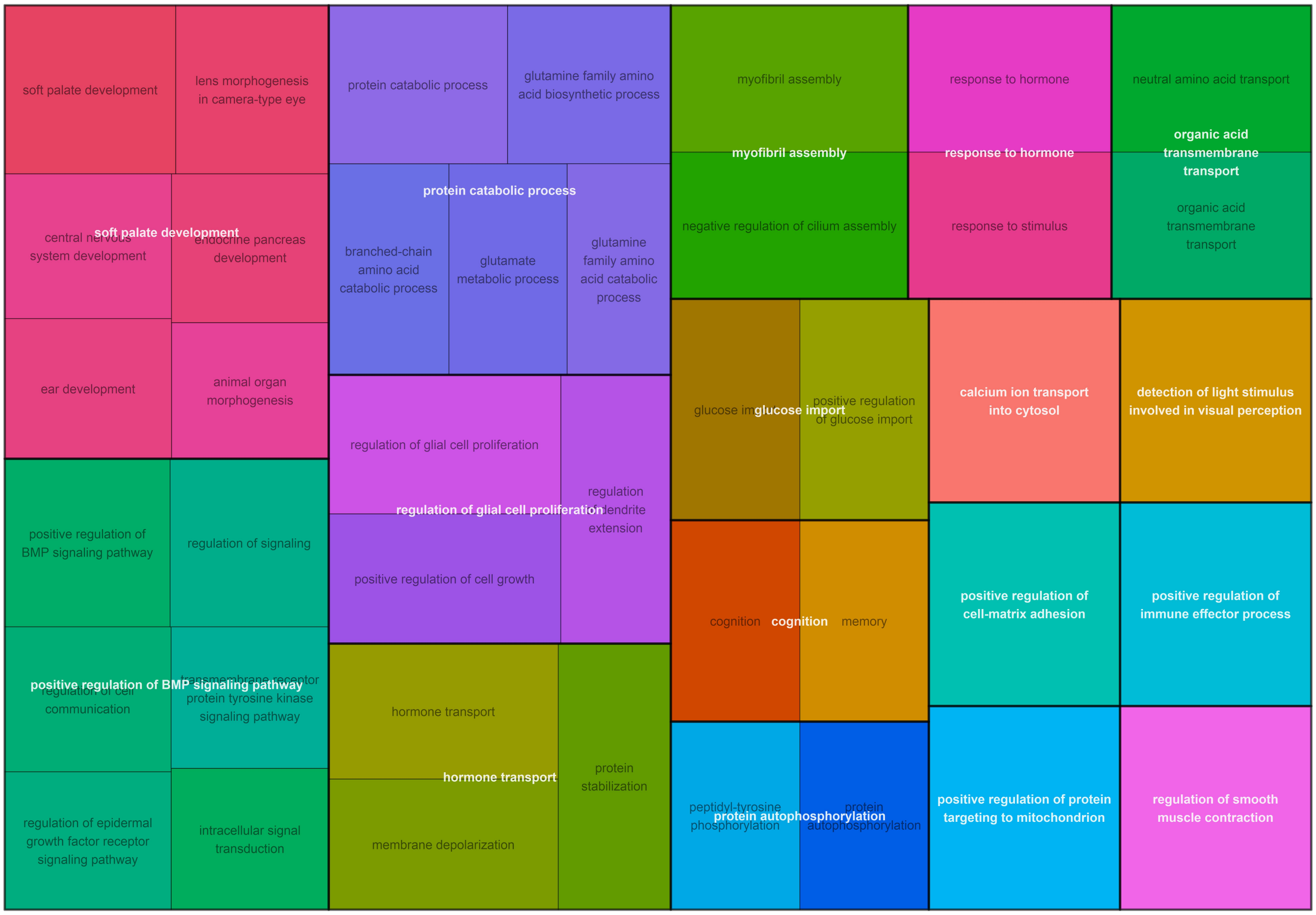

Fig. S3. Cluster 5 treemap from rrvgo reduction and grouping of TopGO enrichment analysis of genes. Genes in this cluster increased expression levels slowly through P_{crit}, rapidly increased expression levels during anoxia, and then decreased in expression below that during normoxia.

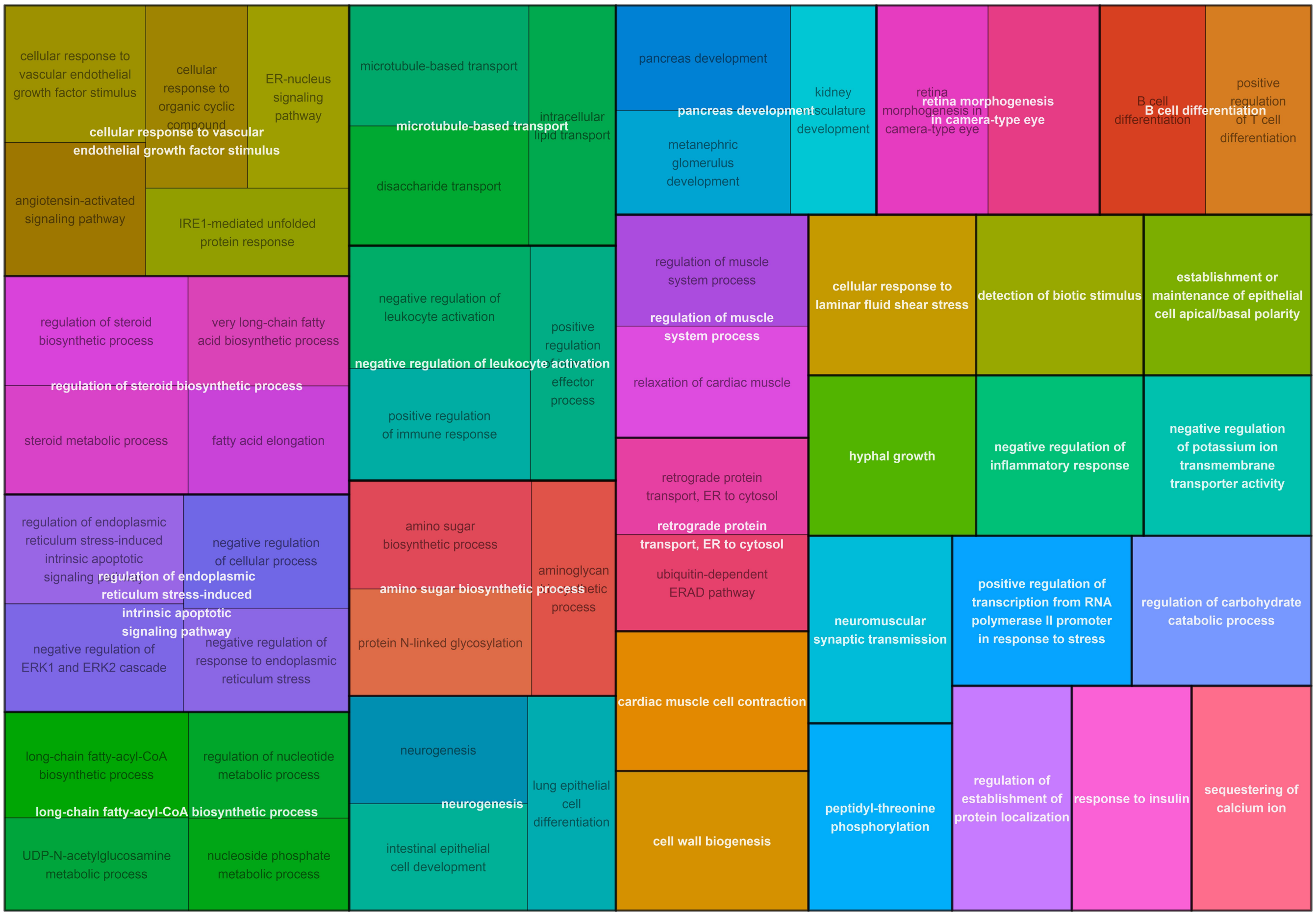

Fig. S4. Cluster 4 treemap from rrvgo reduction and grouping of TopGO enrichment analysis of genes. Genes in this cluster decreased expression levels steadily through anoxia and then increased to pre-stress levels during recovery.

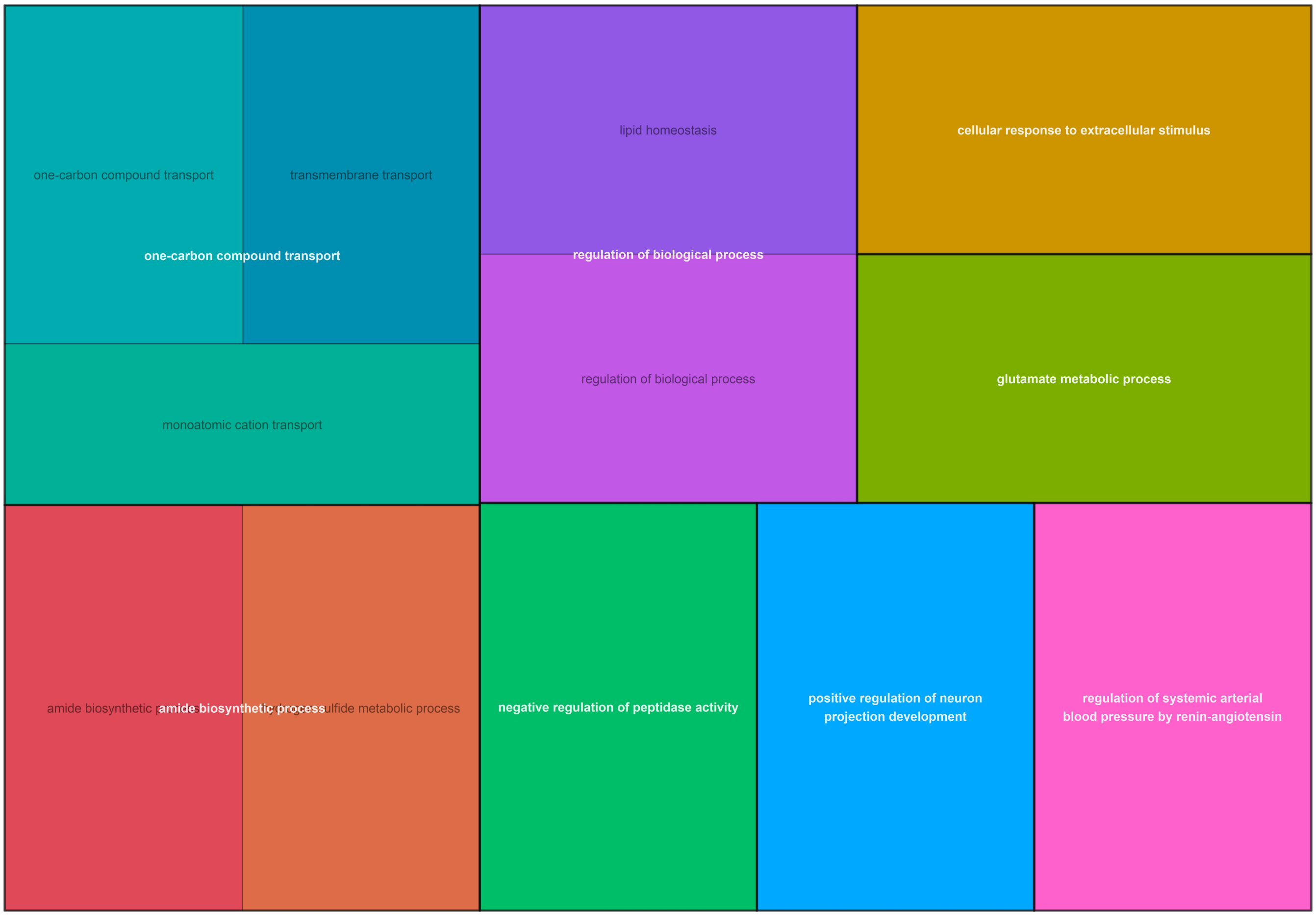

Fig. S5. Cluster 7 treemap from rrvgo reduction and grouping of TopGO enrichment analysis of genes. Genes in this cluster increased expression levels steadily all the way through recovery.

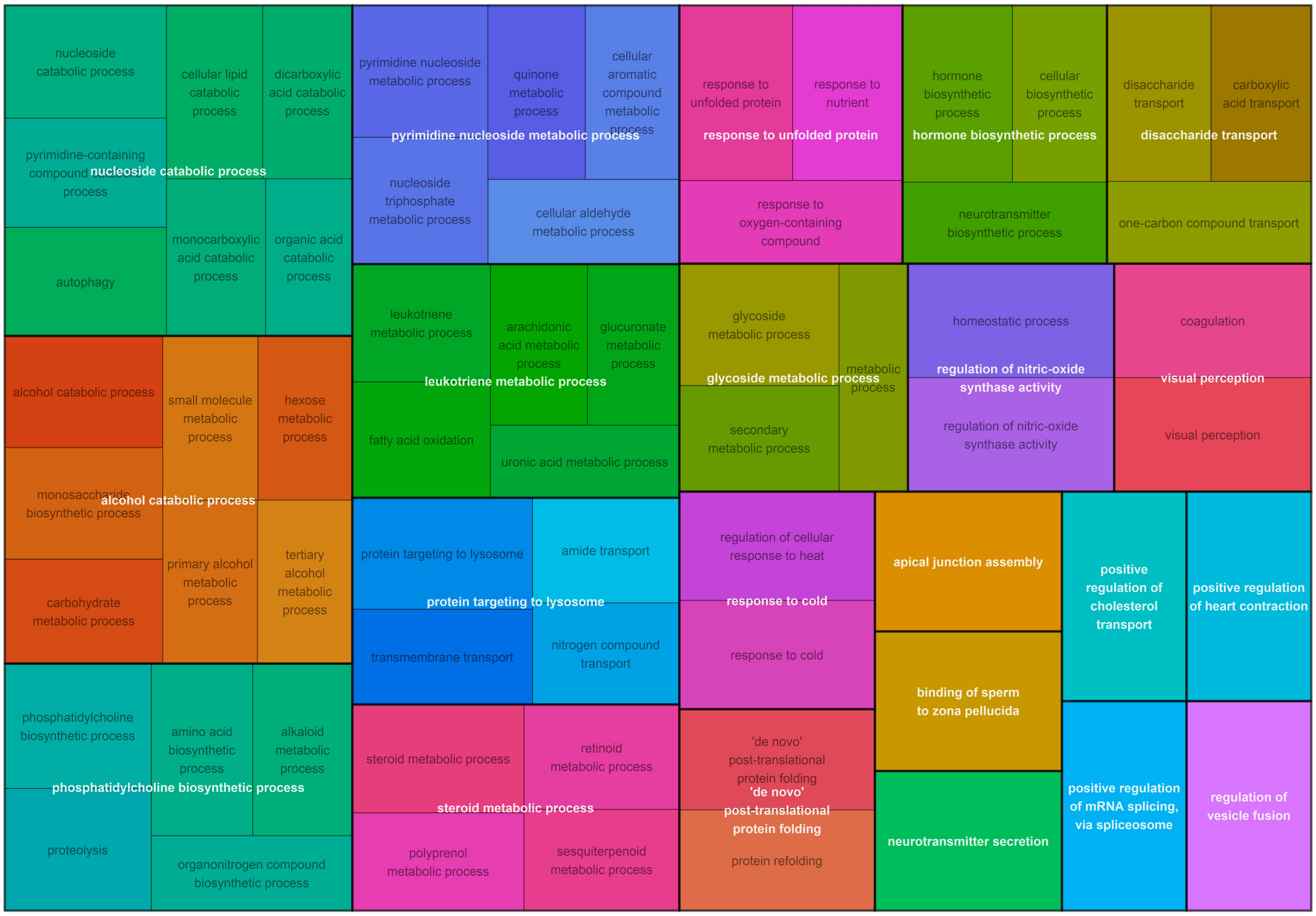

Fig. S6. Cluster 8 treemap from rrvgo reduction and grouping of TopGO enrichment analysis of genes. Genes in this cluster decreased expression levels steadily all the way through recovery.

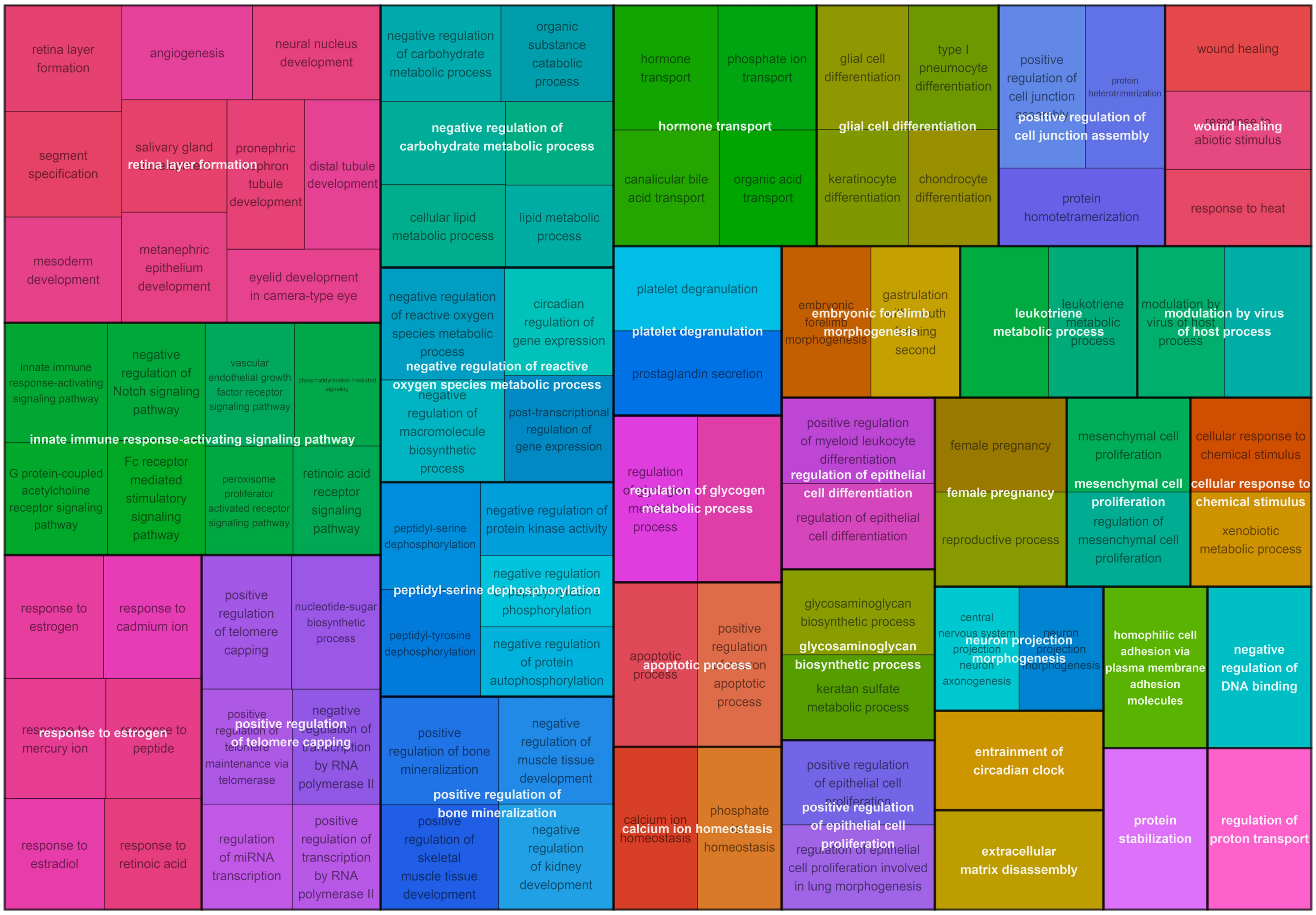

Fig. S7. Cluster 1 treemap from rrvgo reduction and grouping of TopGO enrichment analysis of genes. Genes in this cluster increased expression levels most rapidly when transitioning from anoxia to recovery.

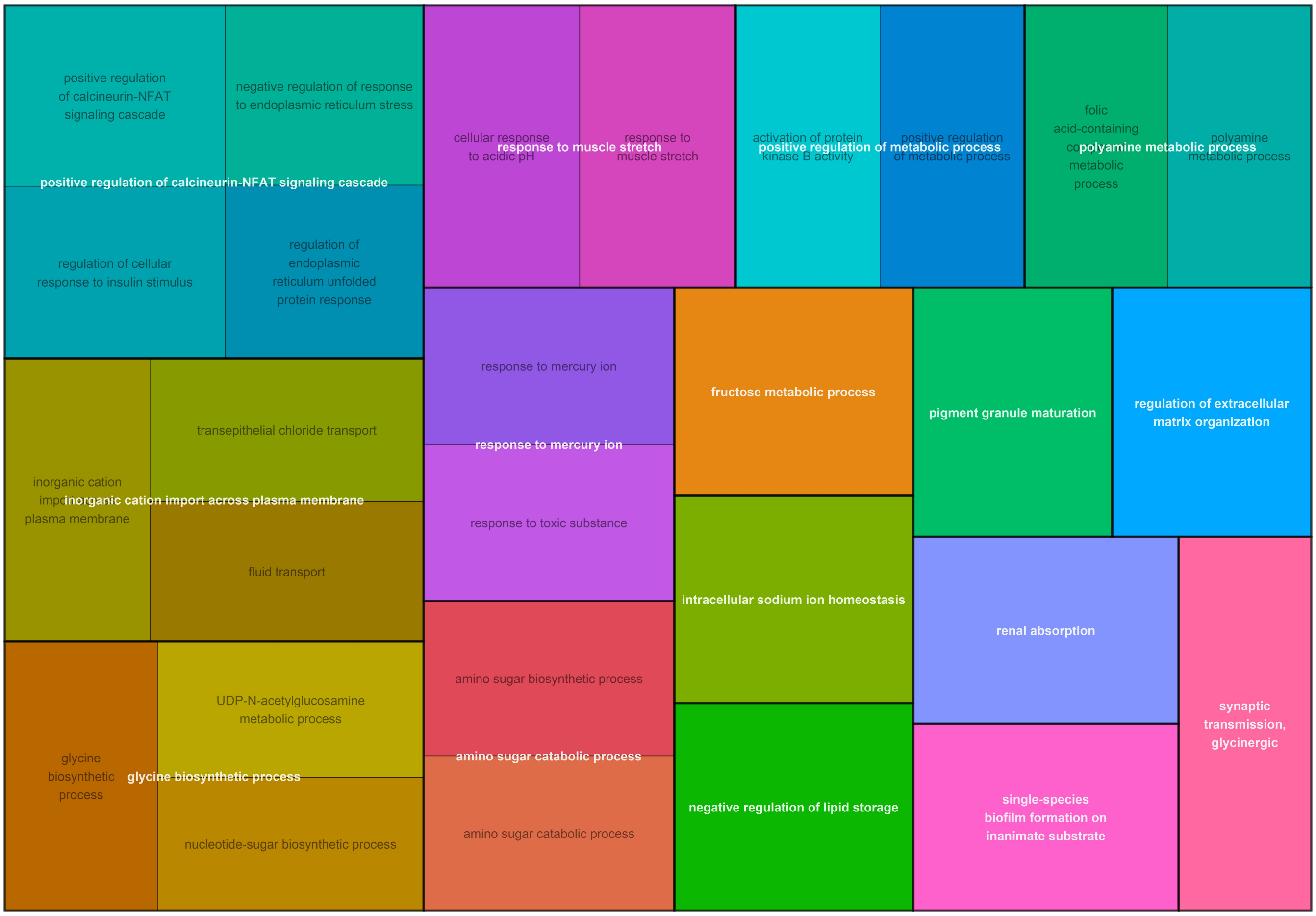

Fig. S8. Cluster 9 treemap from rrvgo reduction and grouping of TopGO enrichment analysis of genes. Genes in this cluster decreased expression levels most drastically when transitioning between anoxia and recovery.

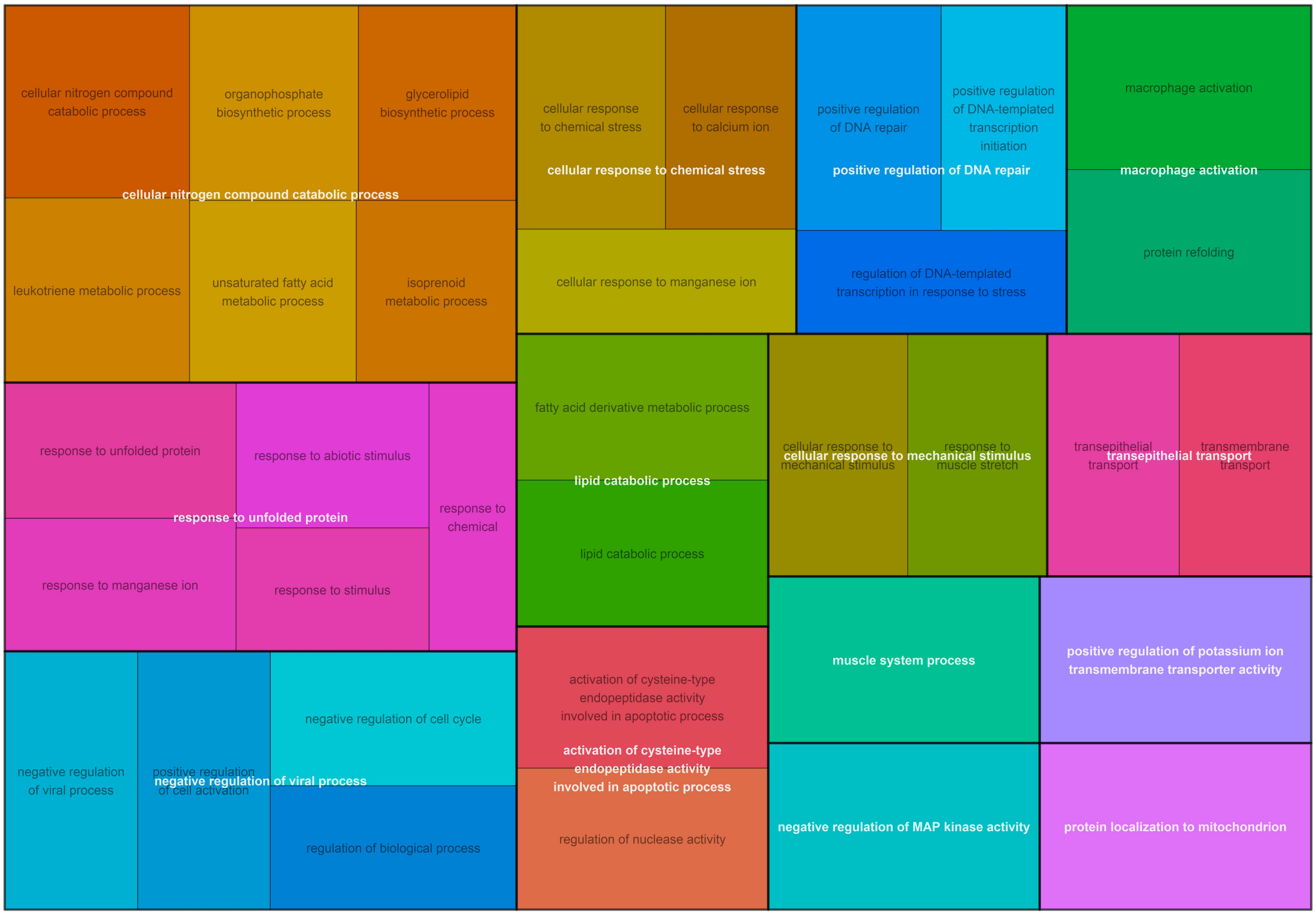

Fig. S9. Cluster 6 treemap from rrvgo reduction and grouping of TopGO enrichment analysis of genes. Genes in this cluster decrased expression levels most drastically at the start of hypoxia exposure and levels remained mostly stable through recovery.

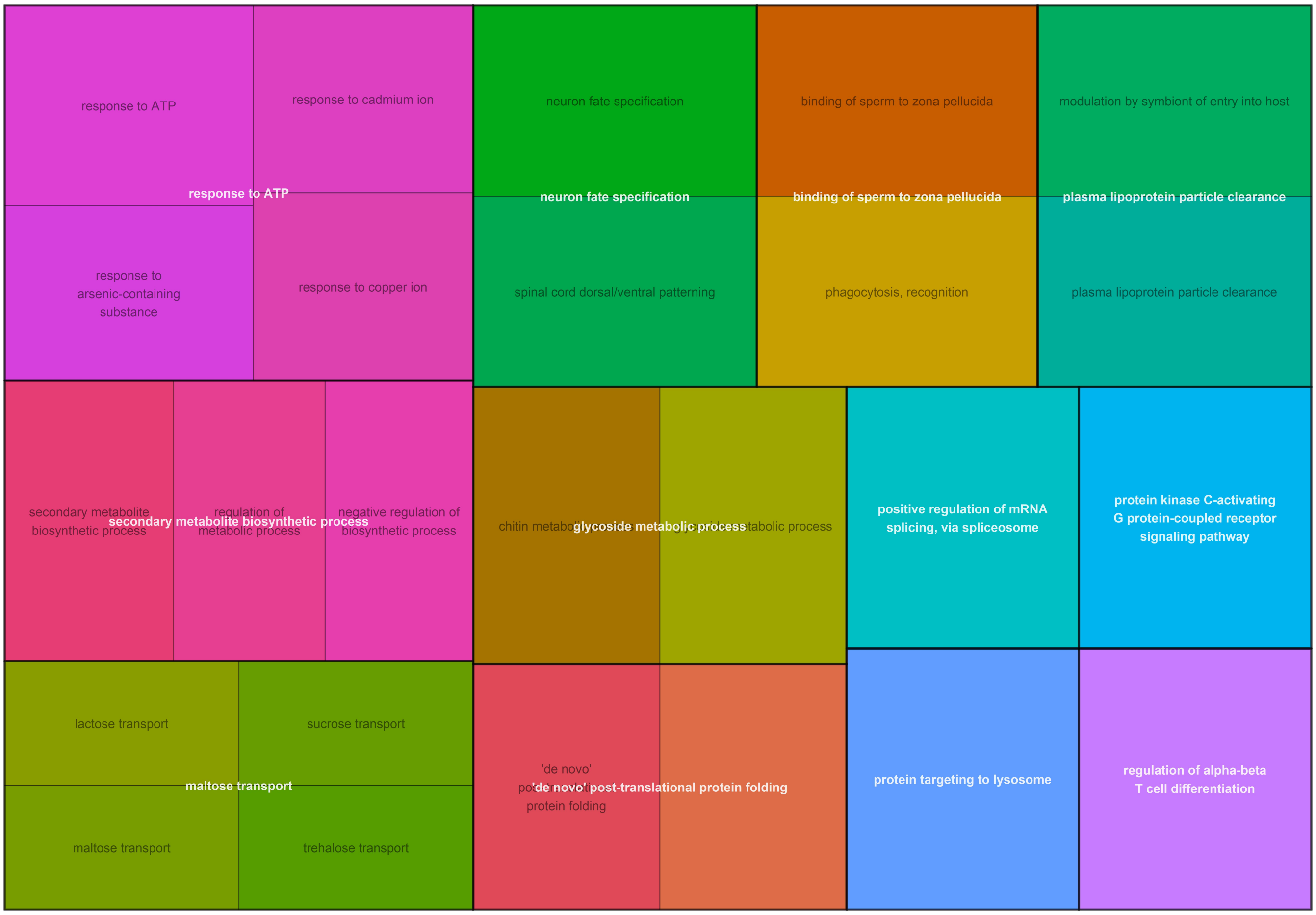

Fig. S10. Treemap from rrvgo reduction and grouping of TopGO enrichment analysis of genes that only showed significant change at the P_{crit} exposure level. Genes in this group increased or decreased expression levels suddenly at P_{crit} and returned to near normoxic levels in anoxia.

Fig. S11. The expression pattern of significant genes identified by maSigPro or DESeq2 in the glycolysis, starch-sucrose, pyruvate, pentose-phosphate, fructose-mannose, TCA cycle, and amino sugar/chitin synthesis pathways. This figure is high resolution and uploaded separate from this document. Details can be read by zooming and scrolling to explore the pathways. Significant genes are highlighted light blue on the pathways. Genes highlighted dark grey are genes listed by NCBI and KEGG as being present in the *T. californicus* genome while genes with no highlighting are not. The heat map of each gene has five segments which correspond to the five time points of the hypoxia course: normoxia, mild hypoxia, hypoxia at P_{crit}, anoxia, and recovery.

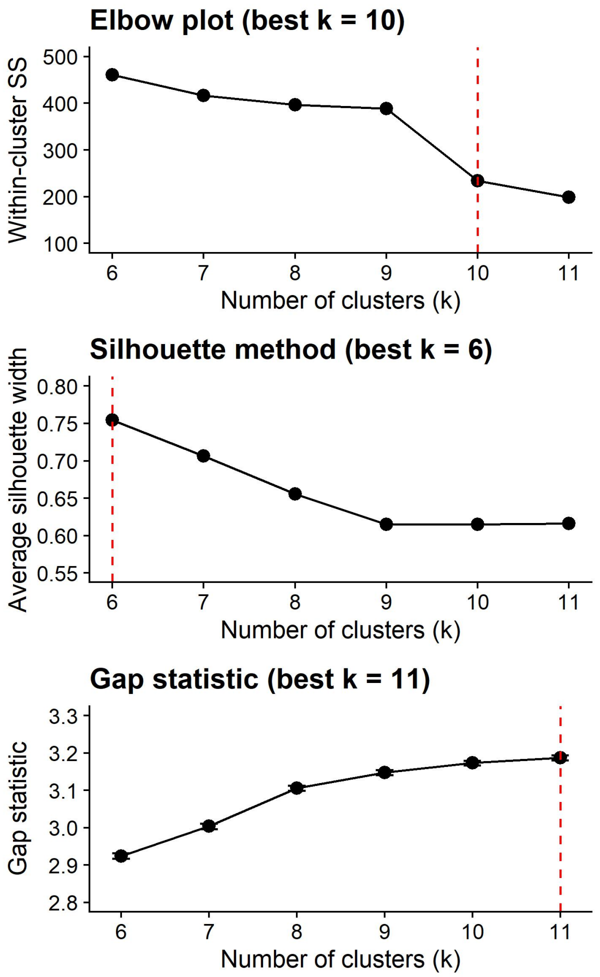

Fig. S12. Clustering statistics for k = 6 through k = 11 clusters. The elbow plot on the top shows the within-cluster sum of squares (wss) and indicates how tightly clusters form at each value of k where lower values indicate lower variation from the cluster center. The "elbow" indicates the point at which the addition of a cluster adds meaningful structure to the groupings. While statistically the addition of a 10th cluster improves the wss, we observed this 10th cluster was visually identical to cluster 2 when k = 9. The silhouette method indicates silhouette width for each cluster and measures cluster separation where lower values indicate better definition. Values above 0.5 are considered to indicate good structure [111]. We observed a width of ~0.63 for our chosen k = 9 clusters. The gap statistic compares clustering to a null distribution that is uniform (i.e., no structure). Larger values indicate improved structure. Increasing the value of k beyond k = 9 showed diminishing improvement, compared to larger increases from k = 6 to k = 9.

References for Supplement Tables S4 and S5

1. Oberhauser, V., Voolstra, O., Bangert, A., von Lintig, J. & Vogt, K. NinaB combines carotenoid oxygenase and retinoid isomerase activity in a single polypeptide. *Proceedings of the National Academy of Sciences* 105, 19000–19005 (2008).

2. Jiang, Z. *et al.* The flavohaemoprotein hmp maintains redox homeostasis in response to reactive oxygen and nitrogen species in Corynebacterium glutamicum. *Microb Cell Fact* 22, 158 (2023).

3. GeneCards. B3GALT1 Gene - Beta-1,3-Galactosyltransferase 1. *GeneCards: The Human Gene Database* https://www.genecards.org/cgi-bin/carddisp.pl?gene=B3GALT1 (2025).

4. Goodspeed, K. *et al.* SLC13A5 Deficiency Disorder: From Genetics to Gene Therapy. *Genes (Basel)* 13, 1655 (2022).

5. Xie, T. *et al.* The Relationship Between HIF1α and Clock Gene Expression in Patients with Obstructive Sleep Apnea. *Nat Sci Sleep* Volume 14, 381–392 (2022).

6. Kaur, A. *et al.* ChaC2, an Enzyme for Slow Turnover of Cytosolic Glutathione. *Journal of Biological Chemistry* 292, 638–651 (2017).

7. Kretzschmar, D. *et al.* Giant lens, a gene involved in cell determination and axon guidance in the visual system of Drosophila melanogaster. *EMBO J* 11, 2531–2539 (1992).

8. Hardin, P. E., Hall, J. C. & Rosbash, M. Feedback of the Drosophila period gene product on circadian cycling of its messenger RNA levels. *Nature* 343, 536–540 (1990).

9. GeneCards. MGST3 Gene - Microsomal Glutathione S-Transferase 3. *GeneCards: The Human Gene Database* https://www.genecards.org/cgi-bin/carddisp.pl?gene=MGST3&keywords=Mgst3 (2025).

10. Leaden, L., Busi, M. V. & Gomez-Casati, D. F. The mitochondrial proteins AtHscB and AtIsu1 involved in Fe–S cluster assembly interact with the Hsp70-type chaperon AtHscA2 and modulate its catalytic activity. *Mitochondrion* 19, 375–381 (2014).

11. Andersen, S. O., Hojrup, P. & Roepstorff, P. Insect cuticular proteins. *Insect Biochem Mol Biol* 25, 153–176 (1995).

12. GeneCards. DDO Gene - D-Aspartate Oxidase. *GeneCards: The Human Gene Database* https://www.genecards.org/cgi-bin/carddisp.pl?gene=DDO&keywords=DDO (2025).

13. House, C. M., Möller, A. & Bowtell, D. D. L. Siah Proteins: Novel Drug Targets in the Ras and Hypoxia Pathways. *Cancer Res* 69, 8835–8838 (2009).

14. Jung, Y.-S. *et al.* TMEM9 promotes intestinal tumorigenesis through vacuolar-ATPase-activated Wnt/β-catenin signalling. *Nat Cell Biol* 20, 1421–1433 (2018).

15. Lee, Y. *et al.* Pyrexia is a new thermal transient receptor potential channel endowing tolerance to high temperatures in Drosophila melanogaster. *Nat Genet* 37, 305–310 (2005).

16. NCBI. Gbs-76A Glycogen binding subunit 76A [Drosophila melanogaster (fruit fly)]. https://www.ncbi.nlm.nih.gov/gene/40102 (2025).

17. McColl, G., Hoffmann, A. A. & McKechnie, S. W. Response of Two Heat Shock Genes to Selection for Knockdown Heat Resistance in *Drosophila melanogaster*. *Genetics* 143, 1615–1627 (1996).

18. Hanington, P. C. & Zhang, S.-M. The Primary Role of Fibrinogen-Related Proteins in Invertebrates Is Defense, Not Coagulation. *J Innate Immun* 3, 17–27 (2011).

19. Jee, C. *et al.* CNP-1 (ARRD-17), a Novel Substrate of Calcineurin, Is Critical for Modulation of Egg-Laying and Locomotion in Response to Food and Lysine Sensation in Caenorhabditis elegans. *J Mol Biol* 417, 165–178 (2012).

20. PomBase. PomBase: SPCC1494.01. https://www.pombase.org/gene/SPCC1494.01 (2025).

21. Hardie, R. C., Martin, F., Chyb, S. & Raghu, P. Rescue of Light Responses in the Drosophila "Null" Phospholipase C Mutant, norpA, by the Diacylglycerol Kinase Mutant, rdgA, and by Metabolic Inhibition. *Journal of Biological Chemistry* 278, 18851–18858 (2003).

22. Scieuzo, C. *et al.* An integrated transcriptomic and proteomic approach to identify the main Torymus sinensis venom components. *Sci Rep* 11, 5032 (2021).

23. Ma, K.-S. *et al.* Identification of microRNAs and their response to the stress of plant allelochemicals in Aphis gossypii (Hemiptera: Aphididae). *BMC Mol Biol* 18, 5 (2017).

24. NCBI. nrf-6 Nose resistant to fluoxetine protein 6 [Caenorhabditis elegans] Gene ID: 174287, updated on 9-Jun-2025 Download Datasets. *NCBI* https://www.ncbi.nlm.nih.gov/gene/174287#:~:text=Nose%20resistant%20to%20fluoxetine%20protein%206%20Nrf-6%20is,activity.%20Predicted%20to%20be%20involved%20in%20lipid%20transport. (2025).

25. GeneCards. SLC12A3 Gene - Solute Carrier Family 12 Member 3. *GeneCards: The Human Gene Database* https://www.genecards.org/cgi-bin/carddisp.pl?gene=SLC12A3 (2025).

26. GeneCards. CYP19A1 Gene - Cytochrome P450 Family 19 Subfamily A Member 1. *GeneCards: The Human Gene Database* https://www.genecards.org/cgi-bin/carddisp.pl?gene=CYP19A1 (2025).

27. Lüersen, K. *et al.* The Glutathione Reductase GSR-1 Determines Stress Tolerance and Longevity in Caenorhabditis elegans. *PLoS One* 8, e60731 (2013).

28. GeneCards. AHCYL2 Gene - Adenosylhomocysteinase Like 2. *GeneCards: The Human Gene Database* https://www.genecards.org/cgi-bin/carddisp.pl?gene=AHCYL2 (2025).

29. GeneCards. UGP2 Gene - UDP-Glucose Pyrophosphorylase 2. *GeneCards: The Human Gene Database* https://www.genecards.org/cgi-bin/carddisp.pl?gene=UGP2 (2025).

30. Kanamori, Y. *et al.* The trehalose transporter 1 gene sequence is conserved in insects and encodes proteins with different kinetic properties involved in trehalose import into peripheral tissues. *Insect Biochem Mol Biol* 40, 30–37 (2010).

31. FlyBase. Gene: Dmel\Ets98B. *FlyBase* https://flybase.org/reports/FBgn0005659.htm (2025).

32. GeneCards. PTCHD3 Gene - Patched Domain Containing 3 (Gene/Pseudogene). *GeneCards: The Human Gene Database* https://www.genecards.org/cgi-bin/carddisp.pl?gene=PTCHD3 (2025).

33. Hartenstein, K. *et al.* The *congested-like tracheae* Gene of *Drosophila melanogaster* Encodes a Member of the Mitochondrial Carrier Family Required for Gas-Filling of the Tracheal System and Expansion of the Wings After Eclosion. *Genetics* 147, 1755–1768 (1997).

34. GeneCards. SIK2 Gene - Salt Inducible Kinase 2. *GeneCards: The Human Gene Database* https://www.genecards.org/cgi-bin/carddisp.pl?gene=SIK2 (2025).

35. GeneCards. GMCL1 Gene - Germ Cell-Less 1, Spermatogenesis Associated. *GeneCards: The Human Gene Database* https://www.genecards.org/cgi-bin/carddisp.pl?gene=GMCL1 (2025).

36. GeneCards. ATXN1 Gene - Ataxin 1. *GeneCards: The Human Gene Database* https://www.genecards.org/cgi-bin/carddisp.pl?gene=ATXN1 (2025).

37. GeneCards. CPPED1 Gene - Calcineurin Like Phosphoesterase Domain Containing 1. *GeneCards: The Human Gene Database* https://www.genecards.org/cgi-bin/carddisp.pl?gene=CPPED1 (2025).

38. Huang, Z. *et al.* Chitin deacetylase: from molecular structure to practical applications. *Systems Microbiology and Biomanufacturing* 2, 271–284 (2022).

39. GeneCards. MUC5AC Gene - Mucin 5AC, Oligomeric Mucus/Gel-Forming. *GeneCards: The Human Gene Database* https://www.genecards.org/cgi-bin/carddisp.pl?gene=MUC5AC (2025).

40. GeneCards. METTL9 Gene - Methyltransferase 9, His-X-His N1(Pi)-Histidine. *GeneCards: The Human Gene Database* https://www.genecards.org/cgi-bin/carddisp.pl?gene=METTL9 (2025).

41. Fujiki, Y. *et al.* Functional identification of *At <scp>AVT</scp> 3* , a family of vacuolar amino acid transporters, in *Arabidopsis*. *FEBS Lett* 591, 5–15 (2017).

42. Jianfeng, W., Yutao, W. & Jianbin, B. TACR2 is associated with the immune microenvironment and inhibits migration and proliferation via the Wnt/β-catenin signaling pathway in prostate cancer. *Cancer Cell Int* 21, 415 (2021).

43. Rajan, M. *et al.* NHR-14 loss of function couples intestinal iron uptake with innate immunity in C. elegans through PQM-1 signaling. *Elife* 8, (2019).

44. GeneCards. SLC18B1 Gene - Solute Carrier Family 18 Member B1. *GeneCards: The Human Gene Database* https://www.genecards.org/cgi-bin/carddisp.pl?gene=SLC18B1 (2025).

45. Lee, S. *et al.* Heat Shock Protein Cognate 70-4 and an E3 Ubiquitin Ligase, CHIP, Mediate Plastid-Destined Precursor Degradation through the Ubiquitin-26S Proteasome System in *Arabidopsis* . *Plant Cell* 21, 3984–4001 (2010).

46. GeneCards. SETD7 Gene - SET Domain Containing 7, Histone Lysine Methyltransferase. *GeneCards: The Human Gene Database* https://www.genecards.org/cgi-bin/carddisp.pl?gene=SETD7 (2025).

47. GeneCards. JPH3 Gene - Junctophilin 3. *GeneCards: The Human Gene Dataset* https://www.genecards.org/cgi-bin/carddisp.pl?gene=JPH3 (2025).

48. GeneCards. SLC6A3 Gene - Solute Carrier Family 6 Member 3. *GeneCards: The Human Gene Dataset* https://www.genecards.org/cgi-bin/carddisp.pl?gene=SLC6A3 (2025).

49. GeneCards. HMCN1 Gene - Hemicentin 1. *GeneCards: The Human Gene Dataset* https://www.genecards.org/cgi-bin/carddisp.pl?gene=HMCN1 (2025).

50. GeneCards. GLS Gene - Glutaminase. *GeneCards: The Human Gene Dataset* GLS Gene - Glutaminase (2025).

51. GeneCards. SPNS2 Gene - SPNS Lysolipid Transporter 2, Sphingosine-1-Phosphate. *GeneCards: The Human Gene Dataset* https://www.genecards.org/cgi-bin/carddisp.pl?gene=SPNS2 (2025).

52. Qiu, X., Hong, H. & MacKenzie, S. L. Identification of a Δ4 Fatty Acid Desaturase fromThraustochytrium sp. Involved in the Biosynthesis of Docosahexanoic Acid by Heterologous Expression inSaccharomyces cerevisiae and Brassica juncea. *Journal of Biological Chemistry* 276, 31561–31566 (2001).

53. Ma, J., Zhen, X., Huang, X. & Jiang, X. Folic acid supplementation repressed hypoxia-induced inflammatory response via ROS and JAK2/STAT3 pathway in human promyelomonocytic cells. *Nutrition Research* 53, 40–50 (2018).

54. UniProt. P08659 · LUCI_PHOPY. *UniProt* https://www.uniprot.org/uniprotkb/P08659/entry#function (2025).

55. GeneCards. CTDSPL2 Gene - CTD Small Phosphatase Like 2. *GeneCards: The Human Gene Dataset* https://www.genecards.org/cgi-bin/carddisp.pl?gene=CTDSPL2 (2025).

56. Nebert, D. W. & Vasiliou, V. Analysis of the glutathione S-transferase (GST) gene family. *Hum Genomics* 1, 460 (2004).

57. López-Olmos, K., Markow, T. A. & Machado, C. A. Evolution of GSTD1 in Cactophilic Drosophila. *J Mol Evol* 84, 285–294 (2017).

58. Park, K. C., Lee, D. C. & Yeom, Y. Il. NDRG3-mediated lactate signaling in hypoxia. *BMB Rep* 48, 301–302 (2015).

59. GeneCards. ATP13A3 Gene - ATPase 13A3. *GeneCards: The Human Gene Database* https://www.genecards.org/cgi-bin/carddisp.pl?gene=ATP13A3 (2025).

60. Kadener, S., Stoleru, D., McDonald, M., Nawathean, P. & Rosbash, M. *Clockwork Orange* is a transcriptional repressor and a new *Drosophila* circadian pacemaker component. *Genes Dev* 21, 1675–1686 (2007).

61. Marco, S. Di & Priestle, J. P. Structure of the complex of leech-derived tryptase inhibitor (LDTI) with trypsin and modeling of the LDTI–tryptase system. *Structure* 5, 1465–1474 (1997).

62. Arakane, Y., Muthukrishnan, S., Beeman, R. W., Kanost, M. R. & Kramer, K. J. *Laccase 2* is the phenoloxidase gene required for beetle cuticle tanning. *Proceedings of the National Academy of Sciences* 102, 11337–11342 (2005).

63. GeneCards. SLC22A1 Gene - Solute Carrier Family 22 Member 1. *GeneCards: The Human Gene Database* https://www.genecards.org/cgi-bin/carddisp.pl?gene=SLC22A1 (2025).

64. Lin, J.-X. *et al.* m6A methylation mediates LHPP acetylation as a tumour aerobic glycolysis suppressor to improve the prognosis of gastric cancer. *Cell Death Dis* 13, 463 (2022).

65. Shanbhag, A. P. FabG: from a core to circumstantial catalyst. *Biotechnol Lett* 41, 675–688 (2019).

66. Marutani, E. *et al.* Sulfide catabolism ameliorates hypoxic brain injury. *Nat Commun* 12, 3108 (2021).

67. Zhang, Y. *et al.* Genetics Responses to Hypoxia and Reoxygenation Stress in Larimichthys crocea Revealed via Transcriptome Analysis and Weighted Gene Co-Expression Network. *Animals* 11, 3021 (2021).

68. ARAL, A. L., ERGÜN, M. A., ENGİN, A. B., BÖRCEK, A. Ö. & BOLAY, H. Iron homeostasis is altered in response to hypoxia and hypothermic preconditioning in brain glial cells. *Turk J Med Sci* 50, 2005–2016 (2020).

69. GeneCards. URAD Gene - Ureidoimidazoline (2-Oxo-4-Hydroxy-4-Carboxy-5-) Decarboxylase. *GeneCards: The Human Gene Database* https://www.genecards.org/cgi-bin/carddisp.pl?gene=URAD (2025).

70. GeneCards. CLPX Gene - Caseinolytic Mitochondrial Matrix Peptidase Chaperone Subunit X. *GeneCards: The Human Gene Database* https://www.genecards.org/cgi-bin/carddisp.pl?gene=CLPX (2025).

71. GeneCards. EHD4 Gene - EH Domain Containing 4. *GeneCards: The Human Gene Database* https://www.genecards.org/cgi-bin/carddisp.pl?gene=EHD4 (2025).

72. Ruff, J., Denger, K. & Cook, A. M. Sulphoacetaldehyde acetyltransferase yields acetyl phosphate: purification from Alcaligenes defragrans and gene clusters in taurine degradation. *Biochemical Journal* 369, 275–285 (2003).

73. Marín, M. *et al.* Modified 3-Oxoadipate Pathway for the Biodegradation of Methylaromatics in *Pseudomonas reinekei* MT1. *J Bacteriol* 192, 1543–1552 (2010).

74. Sailer, S., Keller, M. A., Werner, E. R. & Watschinger, K. The Emerging Physiological Role of AGMO 10 Years after Its Gene Identification. *Life* 11, 88 (2021).

75. Jaunet-Lahary, T. *et al.* Structure and mechanism of oxalate transporter OxlT in an oxalate-degrading bacterium in the gut microbiota. *Nat Commun* 14, 1730 (2023).

76. Islam, R., Brown, S., Taheri, A. & Dumenyo, C. K. The Gene Encoding NAD-Dependent Epimerase/Dehydratase, wcaG, Affects Cell Surface Properties, Virulence, and Extracellular Enzyme Production in the Soft Rot Phytopathogen, Pectobacterium carotovorum. *Microorganisms* 7, 172 (2019).

77. GeneCards. TMEM68 Gene - Transmembrane Protein 68. *GeneCards: The Human Gene Database* https://www.genecards.org/cgi-bin/carddisp.pl?gene=TMEM68 (2025).

78. GeneCards. CD63 Gene - CD63 Molecule. *GeneCards: The Human Gene Database* https://www.genecards.org/cgi-bin/carddisp.pl?gene=CD63 (2025).

79. UniProt. C5J895 · LA1_OPICY. *UniProt* (2025).

80. GeneCards. MDH1 Gene - Malate Dehydrogenase 1. *GeneCards: The Human Gene Database* https://www.genecards.org/cgi-bin/carddisp.pl?gene=MDH1 (2025).

81. NCBI. CG9705 uncharacterized protein [Drosophila melanogaster (fruit fly)]. *NCBI* (2025).

82. PubChem. REPTOR - Repressed by TOR (fruit fly). *NCBI* https://pubchem.ncbi.nlm.nih.gov/gene/REPTOR/fruit_fly (2025).

83. GeneCards. PNKP Gene - Polynucleotide Kinase 3'-Phosphatase. *GeneCards: The Human Gene Database* https://www.genecards.org/cgi-bin/carddisp.pl?gene=PNKP (2025).

84. Paululat, A. *et al.* The Mesodermal Expression of *rolling stone (rost)* Is Essential for Myoblast Fusion in *Drosophila* and Encodes a Potential Transmembrane Protein. *J Cell Biol* 138, 337–348 (1997).

85. Deisl, C. *et al.* Sodium/hydrogen exchanger NHA2 is critical for insulin secretion in β-cells. *Proceedings of the National Academy of Sciences* 110, 10004–10009 (2013).

86. Jung, H. H., Russo, D., Redman, C. & Brandner, S. Kell and XK immunohistochemistry in McLeod myopathy. *Muscle Nerve* 24, 1346–1351 (2001).

87. Wong, J. H. *et al.* Basic Proline-Rich Protein-Mediated Microtubules Are Essential for Lobe Growth and Flattened Cell Geometry. *Plant Physiol* 181, 1535–1551 (2019).

88. GeneCards. SELL Gene - Selectin L. *GeneCards: The Human Gene Database* https://www.genecards.org/cgi-bin/carddisp.pl?gene=sell (2025).

89. GeneCards. MRC2 Gene - Mannose Receptor C-Type 2. *GeneCards: The Human Gene Database* https://www.genecards.org/cgi-bin/carddisp.pl?gene=MRC2 (2025).

90. GeneCards. P4HA1 Gene - Prolyl 4-Hydroxylase Subunit Alpha 1. *GeneCards: The Human Gene Database* https://www.genecards.org/cgi-bin/carddisp.pl?gene=P4HA1 (2025).

91. Andersen, S. O. Studies on resilin-like gene products in insects. *Insect Biochem Mol Biol* 40, 541–551 (2010).

92. GeneCards. SC5D Gene - Sterol-C5-Desaturase. *GeneCards: The Human Gene Database* https://www.genecards.org/cgi-bin/carddisp.pl?gene=SC5D (2025).

93. GeneCards. FUCA1 Gene - Alpha-L-Fucosidase 1. *GeneCards: The Human Gene Database* https://www.genecards.org/cgi-bin/carddisp.pl?gene=FUCA1 (2025).

94. NCBI. LOC135099330 compound eye opsin BCRH2-like [Scylla paramamosain (green mud crab)]. *NCBI* https://www.ncbi.nlm.nih.gov/gene/135099330 (2024).

95. Magkrioti, C. K., Spyropoulos, I. C., Iconomidou, V. A., Willis, J. H. & Hamodrakas, S. J. cuticleDB: a relational database of Arthropod cuticular proteins. *BMC Bioinformatics* 5, 138 (2004).

96. GeneCards. ANKS1A Gene - Ankyrin Repeat And Sterile Alpha Motif Domain Containing 1A. *GeneCards: The Human Gene Database* (2025).

97. Aguilera, J. *et al. The CLAMP GA-Binding Transcription Factor Regulates Heat Stress-Induced Transcriptional Repression by Associating with 3D Chromatin Loops*. (2023) doi:10.1101/2023.10.08.561401.

98. Chopra, V. S. *et al.* Transcriptional activation by GAGA factor is through its direct interaction with dmTAF3. *Dev Biol* 317, 660–670 (2008).

99. Gao, G.-N., Wang, M., Yang, N., Huang, Y. & Xu, R.-M. Structure of Zeste–DNA Complex Reveals a New Modality of DNA Recognition by Homeodomain-Like Proteins. *J Mol Biol* 427, 3824–3833 (2015).

100. Singh, A. *et al.* Stability and dynamics of extradenticle modulates its function. *Curr Res Struct Biol* 7, 100150 (2024).

101. Arredondo, J. J., Vivar, J., Laine-Menéndez, S., Martínez-Morentin, L. & Cervera, M. CF2 transcription factor is involved in the regulation of Mef2 RNA levels, nuclei number and muscle fiber size. *PLoS One* 12, e0179194 (2017).

102. NCBI. pan transcription factor pangolin [Nilaparvata lugens (brown planthopper)]. *NCBI* https://www.ncbi.nlm.nih.gov/gene/?term=LOC111047051 (2024).

103. Sociale, M. *et al.* Ceramide Synthase Schlank Is a Transcriptional Regulator Adapting Gene Expression to Energy Requirements. *Cell Rep* 22, 967–978 (2018).

104. Xin, Z.-T., Carroll, K. A., Kumar, N., Song, K. & Ly, H. Transcriptional Activation of TINF2, a Gene Encoding the Telomere-Associated Protein TIN2, by Sp1 and NF-κB Factors. *PLoS One* 6, e21333 (2011).

105. Ming, Q. *et al.* Structural basis of gene regulation by the Grainyhead/CP2 transcription factor family. *Nucleic Acids Res* 46, 2082–2095 (2018).

106. Meeuse, M. W. M. *et al. C. elegans* molting requires rhythmic accumulation of the Grainyhead/ <scp>LSF</scp> transcription factor <scp>GRH</scp> -1. *EMBO J* 42, (2023).

107. Verzi, M. P., Shin, H., Ho, L.-L., Liu, X. S. & Shivdasani, R. A. Essential and Redundant Functions of Caudal Family Proteins in Activating Adult Intestinal Genes. *Mol Cell Biol* 31, 2026–2039 (2011).

108. Xu, J., Tan, A. & Palli, S. R. The function of nuclear receptors in regulation of female reproduction and embryogenesis in the red flour beetle, Tribolium castaneum. *J Insect Physiol* 56, 1471–1480 (2010).

109. Kim, S., Yu, N.-K. & Kaang, B.-K. CTCF as a multifunctional protein in genome regulation and gene expression. *Exp Mol Med* 47, e166–e166 (2015).

110. Bernard, F., Krejci, A., Housden, B., Adryan, B. & Bray, S. J. Specificity of Notch pathway activation: Twist controls the transcriptional output in adult muscle progenitors. *Development* 137, 2633–2642 (2010).

111. Kaufman, L. & Rousseeuw, P. J. *Finding Groups in Data: An Introduction to Cluster Analysis*. (John Wiley & Sons, Hoboken, New Jersey, 2009).
