# Supplementary figures and images for: Transcriptomic Adjustment to Decreasing Oxygen Reveals Novel Functional Strategies for Extreme Hypoxia Tolerance in the Copepod Tigriopus californicus
Source: Genome Biol Evol. 2026 Feb 11;18(2):evag013. doi: 10.1093/gbe/evag013 (PMC12962235; doi:10.1093/gbe/evag013)

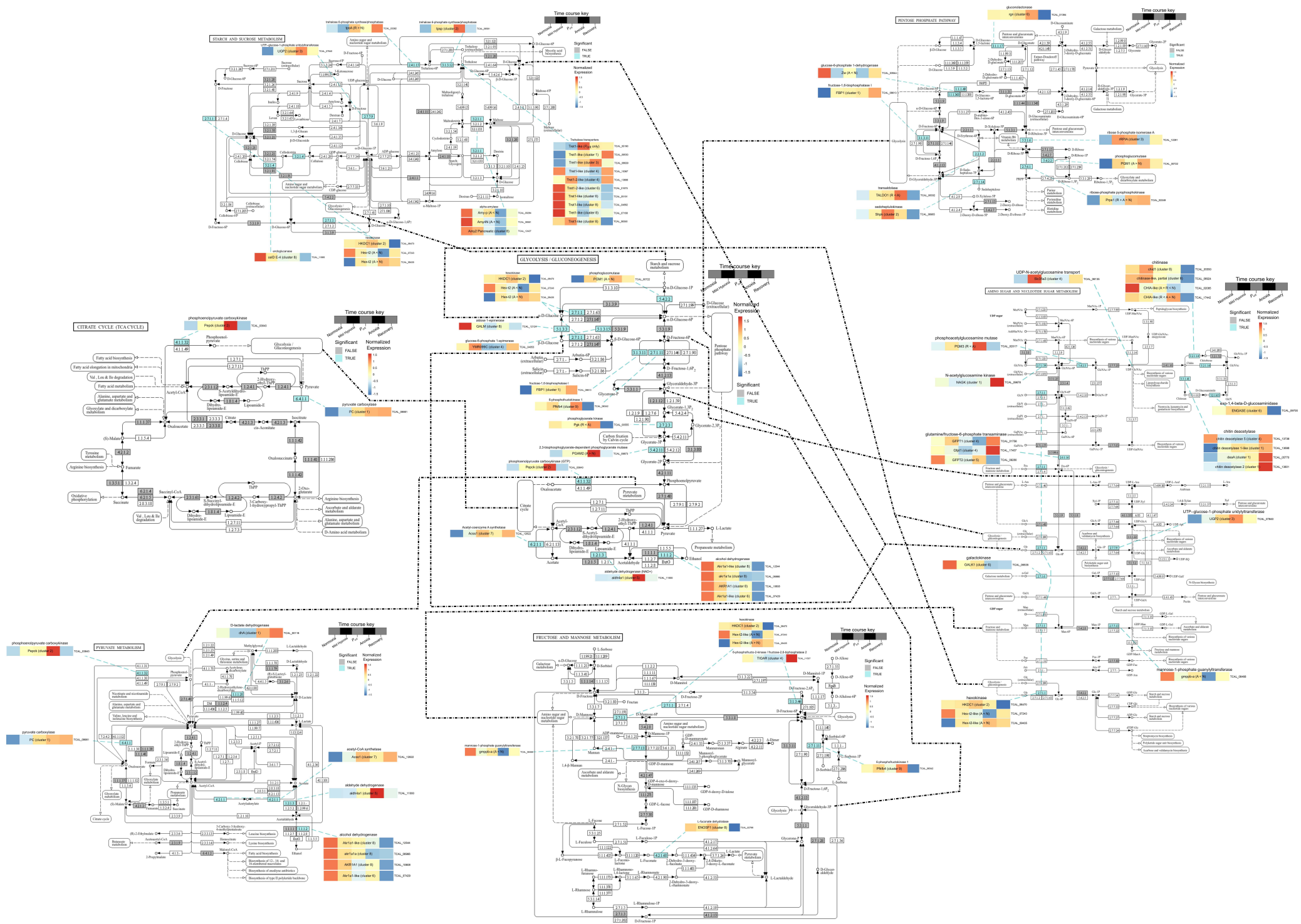

Supplement: evag013_Supplementary_Data [file evag013_supplementary_data.zip › Figure S11.pdf]
